# Developmental Profile of Ion Channel Specializations in the Avian Nucleus Magnocellularis

**DOI:** 10.3389/fncel.2016.00080

**Published:** 2016-03-30

**Authors:** Hui Hong, Lisia Rollman, Brooke Feinstein, Jason Tait Sanchez

**Affiliations:** ^1^Roxelyn and Richard Pepper Department of Communication Sciences and Disorders, The Hugh Knowles Hearing Research Center, School of Communication, Northwestern UniversityEvanston, IL, USA; ^2^Department of Neurobiology and Interdepartmental Neuroscience Program, Weinberg College of Arts and Sciences, Northwestern UniversityEvanston, IL, USA

**Keywords:** action potential, auditory brainstem, development, nucleus magnocellularis, neural excitability, voltage dependent potassium ion channel, voltage dependent sodium ion channel

## Abstract

Ultrafast and temporally precise action potentials (APs) are biophysical specializations of auditory brainstem neurons; properties necessary for encoding sound localization and communication cues. Fundamental to these specializations are voltage dependent potassium (K_V_) and sodium (Na_V_) ion channels. Here, we characterized the functional development of these ion channels and quantified how they shape AP properties in the avian cochlear nucleus magnocellularis (NM). We report that late developing NM neurons (embryonic [E] days 19–21) generate fast APs that reliably phase lock to sinusoidal inputs at 75 Hz. In contrast, early developing neurons (<E12) have slower and less reliable APs that preferentially fire to lower frequencies (5–10 Hz). With development, the membrane time constant of NM neurons became faster, while input resistance and capacitance decreased. Change in input resistance was due to a 2-fold increase in K_V_ current from E10 to E21 and when high-voltage activated potassium (K^+^_HVA_) channels were blocked, APs for all ages became significantly slower. This was most evident for early developing neurons where the ratio of K^+^_HVA_ current accounted for ~85% of the total K_V_ response. This ratio dropped to ~50% for late developing neurons, suggesting a developmental upregulation of low-voltage activated potassium (K^+^_LVA_) channels. Indeed, blockade of K^+^_LVA_ eliminated remaining current and increased neural excitability for late developing neurons. We also report developmental changes in the amplitude, kinetics and voltage dependence of Na_V_ currents. For early developing neurons, increase in Na_V_ current amplitude was due to channel density while channel conductance dominated for late developing neurons. From E10 to E21, Na_V_ channel currents became faster but differed in their voltage dependence; early developing neurons (<E16) had similar Na_V_ channel inactivation voltages while late developing NM neurons (>E19) contained Na_V_ channels that inactivate at more negative voltages, suggesting alterations in Na_V_ channel subtypes. Taken together, our results indicate that the refinement of passive and active ion channel properties operate differentially in order to develop fast and reliable APs in the avian NM.

## Introduction

Auditory temporal processing, or the ability to rapidly encode acoustic information in time, is a specialized ability of auditory brainstem neurons (for review, see Oertel, 1997; Trussell, 1997). It provides the framework for normal and abnormal hearing behaviors (Michalewski et al., 2005) and is critical for survival and communication, such as sound localization and understanding speech in noise (Shannon et al., 1995; Anderson et al., 2010). In order to accurately encode temporal information of sound, auditory brainstem neurons are functionally primed to generate ultrafast and temporally precise action potentials (APs). Such AP properties are conserved in the auditory brainstem of both avians and mammals (Carr et al., 2001). For example, in the avian nucleus laminaris (NL)—and the mammalian analog, the medial superior olive—fast and reliable APs are important for encoding time differences between the two ears on the order of microseconds, cues used to locate sound source within a few degrees along the horizontal plane (Carr and Konishi, 1990; Overholt et al., 1992; Oertel, 1997; Grothe et al., 2010; Köppl, 2012). In order to accomplish this specialized function, neurons from the avian nucleus magnocellularis (NM) and the mammalian analog, the anteroventral cochlear nucleus (AVCN), must provide fast and reliable excitatory inputs to their downstream binaural circuit.

Studies show that passive and active intrinsic properties regulate AP kinetics and precision (Taschenberger and von Gersdorff, 2000; Gao and Lu, 2008). For example, voltage dependent potassium ion channels (K_V_) predominately regulate AP properties in time-coding auditory brainstem neurons (for review, see Johnston et al., 2010). Consistent with this, NM neurons exhibit strong K_V_ conductances composed of two primary subfamilies, Kv1 and Kv3, a low- and high-voltage activated (K^+^_LVA_ and K^+^_HVA_) potassium channel current, respectively (Parameshwaran-Iyer et al., 2001, 2003; Lu et al., 2004; Kuba et al., 2015). K^+^_LVA_ channels open on the slight depolarization of the membrane potential and regulate neural excitability (Rathouz and Trussell, 1998; Howard et al., 2007). Conversely, K^+^_HVA_ channels have higher activation voltages and contribute to rapid AP repolarization and facilitate high frequency firing in mature auditory brainstem neurons (Wang et al., 1998; Klug and Trussell, 2006). However, the specific contribution of K^+^_LVA_ and K^+^_HVA_ channels to AP properties during early development is unknown in NM neurons.

In addition to specific K_V_ channel contributions, studies have documented the functional properties of voltage dependent sodium ion channels (Na_V_) in mature auditory brainstem neurons (Kuba and Ohmori, 2009) and immunohistochemistry studies have shown a shift in Na_V_ channel subtypes as a function of normal development, specifically from Na_V_1.2 to Na_V_1.6-containing channels (Kuba et al., 2014). Interestingly, these different Na_V_ channel subtypes present with distinct voltage dependent properties (e.g., activation and inactivation) in mature spinal sensory neurons (Rush et al., 2005). These findings suggest that Na_V_ channel subtypes may contribute to functional changes in activation and inactivation kinetics that ultimately give rise to fast AP properties. However, whether there is a shift in Na_V_ channel subtypes in NM, as well as the corresponding functional development of Na_V_ channel currents and voltage dependence is largely unexplored in developing NM.

Regardless of these gaps in knowledge, it is clear that the development of specialized intrinsic properties is correlated with changes in spontaneous and evoked afferent activity (for review, see Wang and Bergles, 2015). With respect to the avian system, this occurs largely during a time period when endogenous signaling ceases and the peripheral system “senses” ambient acoustic sound (Jones et al., 2006). Although peripheral spontaneous activity is prominent during an early “prehearing period”, it is not clear how NM neurons are primed for such activity early in development. In this study, we characterized the developmental profile of intrinsic ion channel properties at functionally distinct developmental time periods, corresponding to before, during and after the onset of hearing in the avian NM.

We report that the membrane time constant, capacitance and input resistance decrease with development, enhancing AP speed. The decrease in input resistance corresponds to a significant upregulation of K_V_ channels, further shaping AP properties and establishing a mature-like AP firing phenotype. In particular, K^+^_HVA_ channels regulate AP kinetics while K^+^_LVA_ control neural excitability. The strength of these regulations varies at different ages due to developmental differences in K_V_ channel subtypes. Similarly, Na_V_ channel current properties and voltage dependence dramatically change, suggesting a switch in Na_V_ channel subtypes. Taken together, the above interaction of passive and active ion channel specializations promotes the maturation of ultrafast and temporally precise APs in the avian NM.

## Materials and Methods

### Slice Preparation

All animal procedures were performed in accordance with federal guidelines on animal welfare and approved by Northwestern University Institutional Animal Care and Use Committee. Acute brainstem slices were prepared from White Leghorn chicken (*Gallus gallus domesticus*) embryos as previously described (Sanchez et al., 2010, 2011, 2012, 2015). Developmental ages for the current study were embryonic days (E) 10–12, 14–16, 19–21, corresponding to before, during, and after hearing onset, respectively (Jones et al., 2006). Briefly, the brainstem was dissected and isolated in ice-cold (~0^°^C) oxygenated low-Ca^2+^ high-Mg^2+^ modified artificial cerebral spinal fluid (ACSF) containing the following (in mM): 130 NaCl, 2.5 KCl, 1.25 NaH_2_PO_4_, 26 NaHCO_3_, 3 MgCl_2_, 1 CaCl_2_, and 10 glucose. ACSF was continuously bubbled with a mixture of 95% O_2_/5% CO_2_ (pH 7.4, osmolarity 295–310 mOsm/l). The brainstem was blocked coronally, affixed to the stage of a vibratome slicing chamber (Ted Pella, Inc., Redding, CA, USA) and submerged in ice-cold ACSF. Bilaterally symmetrical coronal slices were made (200–300 μm thick) and approximately 3–6 slices (depending on age) containing NM were taken from caudal to rostral, roughly representing the low-to-high frequency regions, respectively. All neurons reported here were obtained from the rostral one-half of the entire nucleus, roughly representing the mid-to-high frequency regions of NM.

Slices were collected in a custom holding chamber and allowed to equilibrate for 1 h at ~22^°^C in normal ACSF containing the following (in mM): 130 NaCl, 2.5 KCl, 1.25 NaH_2_PO_4_, 26 NaHCO_3_, 1 MgCl_2_, 3 CaCl_2_, and 10 glucose. For a subset of experiments, recordings were obtained at near physiological temperatures (~35^°^C, see “Results” Section). Normal ACSF was continuously bubbled with a mixture of 95% O_2_/5% CO_2_ (pH 7.4, osmolarity 295–310 mOsm/l). Slices were transferred to a recording chamber mounted on an Olympus BX51W1 (Center Valley, PA, USA) microscope for electrophysiological experiments. The microscope was equipped with a CCD camera, 60× water-immersion objective and infrared differential interference contrast optics. The recording chamber was superfused continuously (Welco, Tokyo, Japan) at room or near physiological temperatures (monitored continuously at either ~22^°^or ~35^°^C, Warner Instruments, Hamden, CT, USA) in oxygenated normal ACSF at a rate of 1.5–2 ml/min.

### Whole Cell Electrophysiology

Voltage-clamp and current-clamp experiments were performed using an Axon Multiclamp 700B amplifier (Molecular Devices, Silicon Valley, CA, USA). Patch pipettes were pulled to a tip diameter of 1–2 μm using a P-97 flaming/brown micropipette puller (Sutter Instrument, Novato, CA, USA) and had resistances ranging from 2 to 6 MΩ. For voltage-clamp experiments of isolated K_V_ currents, the internal solution contained the following (in mM): 105 K-gluconate, 35 KCl, 1 MgCl_2_, 10 HEPES-K^+^, 5 EGTA, 4 4-ATP-Mg^2+^, 0.3 4-Tris2GTP, pH adjusted to 7.3–7.4 with KOH. The junction potential was ~−10 mV and data were corrected accordingly. For voltage-clamp experiments of isolated Na_V_ currents, the internal solution was cesium-based and contained the following (in mM): 150 CsCl, 10 NaCl, 0.2 EGTA, 10 HEPES, pH adjusted to 7.3–7.4 with CsOH. The junction potential was ~−3 mV and data were not corrected. The Cs-based internal solution was used to block K_V_ currents and reduce space-clamp issues. Series resistance was compensated for by ~80% in all voltage-clamp recordings. For current-clamp experiments, the internal solution was potassium-based and contained the following (in mM): 105 K-gluconate, 35 KCl, 1 MgCl_2_, 10 HEPES-K^+^, 5 EGTA, 4 4-ATP-Mg^2+^, and 0.3 4-Tris2GTP, pH adjusted to 7.3–7.4 with KOH. The junction potential was not corrected for in our current clamp experiments.

A small hyperpolarizing (−1 mV, 30 ms) voltage command was presented at the beginning of each recorded trace to document and monitor whole-cell parameters (resting membrane potential [RMP], cell membrane capacitance, series resistance and input resistance). Neurons were included in the data analysis only if they had RMPs more negative than −45 mV and had series resistances <15 MΩ. Raw data was low-pass filtered at 2 kHz and digitized at 20 kHz using a Digidata 1440A (Molecular Devices). For a subset of current and voltage clamp experiments, data was low-pass filtered at 5 kHz and digitized at 50 kHz for NM neurons at E10–12 and E19–21. It should be noted that the lower low-pass filter frequency (2 kHz) underestimated the actual kinetics of AP and Na_V_ current for neurons at E19–21. As for sampling rate, the developmental trends we report were similar between the two sampling rates and thus data obtained under these two rates were shown together (see “Results” Section).

Pipettes were visually guided to NM and neurons were identified and distinguished from surrounding tissue based on cell morphology, known structure, and location of the nucleus within the slice. All experiments were conducted in the presence of a GABA_A_-R antagonist picrotoxin (PTX, 100 μM). After a GΩ seal was attained, membrane patches were ruptured and NM neurons were held in whole-cell configuration for voltage-clamp recordings at membrane potentials ranging between −90 mV to +30 mV. For both isolated K_V_ and Na_V_ voltage-clamp experiments, synaptic glutamate transmission was continuously blocked using DL-2-amino-5-phosphonopentanoic acid (DL-APV, 100 μM, an NMDA-R receptor antagonist) and 6-Cyano-7-nitroquinoxaline-2, 3-dione (CNQX, 20 μM, an AMPA-R receptor antagonist). Isolated K_V_ currents were recorded in the presence of the Na_V_ blocker tetrodotoxin (TTX, 1 μM) and isolated Na_V_ currents were recorded with bath application of K_V_ channel blockers tetraethylammonium (TEA, 3 mM) and (4-AP, 30 μM). In a subset of experiments for recording isolated Na_V_ currents, external Na^+^ concentration levels were reduced to 72 mM by isotonic replacement with TEA (Kuba and Ohmori, 2009). Additionally, 4-AP (30 μM) and Cs^+^ (5 mM) were added to the bath solution. Na_V_ current recordings made in normal and low-Na^+^ ACSF were reported separately (see “Results” Section). Fluoxetine (Flx, 100 μM), a highly potent blocker of Kv3.1-containing K^+^_HVA_ channels (Sung et al., 2008), was bath applied to estimate the ratio of K^+^_LVA_ and K^+^_HVA_ currents. Dendrotoxin (DTx, 0.1 μM), a potent blocker of Kv1.1, Kv1.2-containing K^+^_LVA_ channels, was bath applied to characterize the function of K^+^_LVA_ channels. Potassium leak currents were measured offline using the averaged responses to hyperpolarizing steps from −80 to −90 mV as a baseline and were subtracted from the raw data.

Under whole-cell current clamp, we first characterized the passive intrinsic properties for each age group by injecting a small hyperpolarizing current into the soma (−10 pA, Franzen et al., 2015). This paradigm minimizes the recruitment of voltage dependent ion channels that are not active at or near rest. Membrane voltages used for data analysis were averaged over 30 repetitive trials and calculated by fitting a single exponential to the first 10 ms time window following the hyperpolarizing current injection. The membrane input resistance (R_M_) was obtained by dividing the calculated steady-state membrane voltage by the injected current. The time constant of the membrane voltage (tau, T_M_) was quantified by fitting a single exponential as described above and membrane capacitance (C_M_) was calculated as C_M_ = T_M_/R_M_.

K_V_ conductances (*G*_k_) and Na_V_ conductances (*G*_Na_) were obtained by the equation *I*_k/Na_ = *G*_k/Na_ (*V*_MEMBRANE_ − *E*_k/Na_). *I*_k_ represents the potassium current measured in response to membrane voltage (*V*_MEMBRANE_). Based on our external and internal recording solutions, the reversal potential for K_V_ (*E*_k_) was −84 mV. *G*_Na_ was obtained by the same equation as listed above. The reversal potential for Na_V_ channels was taken from the experimental data for each individual neuron. For both K_V_ and Na_V_, channel density was calculated by normalizing isolated currents to the individual membrane capacitance. Na_V_ channel inactivation (*h*_Na_) curves were fit using a Boltzmann-type function where $h_{\text{Na}}\;=\;1/[1+e^{(V_{\text{MEMBRANE}}-V_{1\text{/2}})/k}]$, in order to calculate half inactivation voltage (*V*_1/2_).

For current-clamp experiments, NM neurons were held in whole-cell configuration at *I* = 0 for recording intrinsic properties. The amount of current needed to evoke an AP developmentally changes as a function of maturation. In order to compare across different age groups, we measured the AP threshold current for each individual neuron. AP threshold current is defined as the minimum amount of current required for NM neurons to generate an AP ~50% of the time across 30 repetitive stimulations (interpulse stimulus intervals = 2 s). Once AP threshold current was obtained, a sustained current command (duration = 100 ms) was injected into the soma at 25% above the measured threshold current for each neuron. APs evoked by this current command were used to characterize AP properties.

### Data Analysis

Recording protocols were written and run using Clampex acquisition and Clampfit analysis Software (version 10.3; Molecular Devices, Silicon Valley, CA, USA). Statistical analyses and graphing protocols were performed using Prism (GraphPad versions 6.07, La Jolla, CA, USA) and MATLAB (version R2014b; The Math Works, Natick, MA, USA) Software. Analysis of variance and *post hoc* Bonferroni adjusted *t*-tests were used to determine significance. The standard for significant differences was defined as *p* < 0.05. All graphic representations of data illustrate individual neurons and bars represent the mean. Data shown in Tables represent mean ± 1 standard deviation.

### Reagents

All bath applied drugs were allowed to perfuse through the recording chamber for ~10 min before subsequent recordings. DL-APV, CNQX and all other salts and chemicals were obtained from Sigma-Aldrich (St. Louis, MO, USA). PTX and Flx were obtained from Tocris (Ellisville, MO, USA). TTX and DTx were obtained from Alomone Labs (Jerusalem, Israel). TEA was obtained from VWR (Radnor, PA, USA).

## Results

The result reported here are from a total of 251 NM neurons obtained from embryos at E10–12, E14–16 and E19–21. These developmental time periods correspond to before, during and after the onset of hearing in chickens, respectively (Jones et al., 2006). Using whole-cell current clamp methods, we characterized the development of active and passive membrane properties in response to somatic current injections. Using whole-cell voltage clamp methods, we characterized the development of K_V_ and Na_V_ channel properties.

### Development of Active Membrane Properties in NM

**Figure 1:**
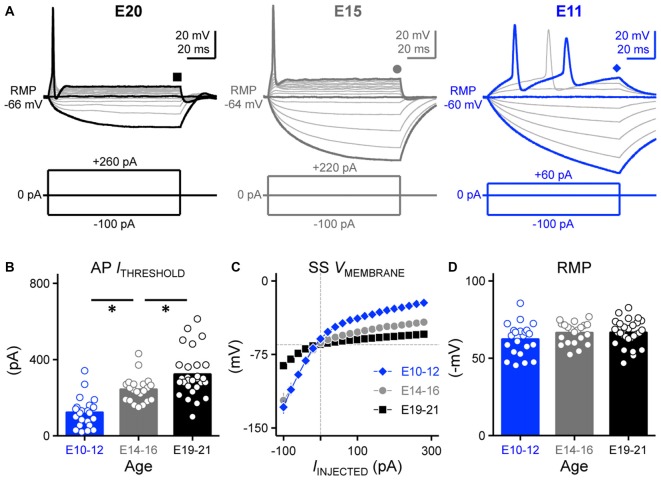
**Developing nucleus magnocellularis (NM) neurons showed distinct firing patterns in response to sustained current injections. (A)** Representative voltage traces recorded from NM neurons at E20 (*left*), E15 (*middle*) and E11 (*right*) in response to a sequence of sustained current injections shown below the traces (current step = 20 pA, current duration = 100 ms). Symbols (square, circle and diamond) at the end of voltage trace represent the time window of voltage measured and plotted as a function of injected current shown in **(C)**. Schematic representation of stimuli used to evoke responses are shown below the representative traces. **(B)** Population data showing threshold current for AP generation as a function of age. **(C)** Population data showing the voltage-current relationship for each age group. **(D)** Population data showing the resting membrane potential (RMP) as a function of age. Open circles represent an individual neuron and solid bars represent the average for each age group. Error bars = standard error. *p < 0.05, Bonferroni adjusted *t*-test.

A biophysical hallmark of many time-coding auditory brainstem neurons is the generation of a single onset AP in response to a sustained depolarizing somatic current injection (Oertel, 1983; Reyes et al., 1994). Figure 1A (*left*) shows such a response from an E20 NM neuron to a series of current injections, the largest of which was the threshold current for AP generation for this neuron. When depolarized with long-duration current steps, all late developing neurons (E19–21) fired only a single AP at the onset of the current step and maintained a stable membrane voltage following the initial AP. In no case did E19–21 neurons fire multiple APs to threshold and suprathreshold current steps, a stereotypical phenotype of late developing NM neurons.

We characterized this response property in early developing neurons. By E14–16, all recorded neurons fired only a single onset AP to sustained depolarization, similar to late developing neurons (21 of 21 neurons). However, lower threshold currents were sufficient to elicit the single AP response (Figure 1A, *middle*). This lower threshold current was also evident for neurons at E10–12 (Figure 1A, *right*) and across the population of neurons tested, the earliest developing neurons (E10–12) had the lowest threshold currents needed to elicit APs compared to E14–16 and E19–21 neurons (*p* < 0.0001, Figure 1B, Table 1). For a subset of E10–12 neurons (16 of 22), suprathreshold current injection resulted in multiple AP generation during the duration of the current step.

**Table 1:** **Maturation of membrane, action potential, potassium current and sodium current properties**.

	E10–E12	E14–E16	E19–E21	ANOVA *P*
**Membrane properties**
RMP (mV)	−62.21 ± 10.31 (22)	−66.33 ± 6.87 (21)	−66.52 ± 8.49 (28)	*P* = 0.17 (Figure 1D)
Time constant tau (ms)	15.57 ± 6.23 (10)	5.90 ± 3.26 (13)	3.18 ± 1.33 (20)	*P* < 0.0001 (Figure 5B)
Input resistance (MΩ)	324.60 ± 120.30 (10)	211.40 ± 49.87 (13)	123.90 ± 49.90 (20)	*P* < 0.0001 (Figure 5C)
Cell capacitance (pF)	47.91 ± 4.61 (10)	26.78 ± 9.97 (13)	26.15 ± 4.60 (20)	*P* < 0.0001 (Figure 5D)
**Action potential (AP) properties**
Threshold current (pA)	121.30 ± 80.30 (22)	242.70 ± 68.58 (21)	321.70 ± 121.00 (28)	*P* < 0.0001 (Figure 1B)
Latency (ms)	19.49 ± 10.86 (22)	4.17 ± 1.15 (21)	3.00 ± 0.57 (28)	*P* < 0.0001 (Figure 2C)
Max rise rate (mV/ms)	38.59 ± 12.13 (22)	98.76 ± 40.98 (21)	155.60 ± 42.19 (28)	*P* < 0.0001 (Figure 2E)
Max fall rate (mV/ms)	−19.26 ± 5.78 (22)	−56.30 ± 25.85 (21)	−104.40 ± 29.79 (28)	*P* < 0.0001 (Figure 2F)
AP half width (ms)	4.57 ± 1.07 (21)	1.91 ± 0.93 (21)	0.97 ± 0.17 (28)	*P* < 0.0001 (Figure 2D)
AP reliability range (ms)	8.89 ± 8.98 (22)	0.44 ± 0.24 (21)	0.21 ± 0.14 (28)	*P* < 0.0001 (Figure 3C)
AP height (mV)^§^	69.09 ± 11.64 (22)	80.27 ± 8.83 (21)	82.94 ± 9.97 (28)	*P* < 0.0001
**K_V_ current (*I*_k_)**
Total *I*_k_ at +20 mV (pA)	3076 ± 1272 (35)	4386 ± 1105 (42)	6240 ± 1327 (39)	*P* < 0.0001 (Figure 6B)
Total *I*_k_ conductance (nS)	26.99 ± 11.16 (35)	38.47 ± 9.70 (42)	54.74 ± 11.64 (39)	*P* < 0.0001 (Figure 6C)
Total *I*_k_ density (pA/pF)	71.71 ± 26.75 (10)	172.00 ± 65.47 (13)	258.60 ± 65.62 (15)	*P* < 0.0001 (Figure 6D)
**Na_V_ current (*I*_Na_)**
V_25%_ (mV)^∧^	−33.64 ± 4.99 (14)	−40.50 ± 5.57 (24)	−50.36 ± 6.02 (11)	*P* < 0.0001
*I*_Na_ (pA)*	−1728.00 ± 759.10 (14)	−2453.00 ± 654.00 (24)	−3386.00 ± 1089.00 (11)	*P* < 0.0001 (Figure 10B)
*I*_Na_ conductance (nS)*	24.15 ± 8.90 (14)	32.43 ± 7.51 (24)	44.97 ± 12.07 (11)	*P* < 0.0001 (Figure 11C)
*I*_Na_ density (pA/pF)*	−36.17 ± 13.99 (12)	−84.16 ± 48.52 (20)	−98.59 ± 53.48 (10)	*P* < 0.01 (Figure 11D)
Max rise rate (pA/ms)*	−4223.00 ± 2906.00 (14)	−7836.00 ± 3837.00 (24)	−10546.00 ± 5736.00 (11)	*P* < 0.01 (Figure 10C)
Max fall rate (pA/ms)*	1008.00 ± 735.00 (14)	1842.00 ± 776.80 (24)	2782.00 ± 1117.00 (11)	*P* < 0.0001 (Figure 10D)
Half width (ms)*	1.99 ± 1.03 (14)	1.29 ± 0.37 (24)	1.13 ± 0.14 (11)	*P* < 0.01 (Figure 10E)
Reliability range (ms)*	0.46 ± 0.42 (22)	0.39 ± 0.39 (23)	0.50 ± 0.42 (18)	*P* = 0.69 (Figure 10F)
*V*_1/2_ (mV)	−49.87 ± 3.20 (7)	−47.13 ± 5.27 (8)	−54.67 ± 3.77 (7)	*P* < 0.01 (Figure 11F)

RMP, Resting membrane potential. ^§^Peak AP amplitude measured relative to the RMP. ^∧^Holding voltage 25% above activation voltage. *Measured at V_25%_ for each developmental age.

The steady-state membrane voltage shows that late developing NM neurons (E19–21) have the lowest input resistance compared to early developing neurons (Figure 1C, Table 1). The voltage-current relationships show that the greatest amount of change in the steady-state membrane voltage was observed above and below the average RMPs between E10–12 and E19–21 neurons. However, the average RMPs across the entire population of neurons tested for each age group were not significantly different (*p* = 0.17, Figure 1D, Table 1), suggesting that the decrease in rectification above the RMP of late developing neurons is likely due to a increase in outward K_V_ conductances (see below).

### Development of AP Properties in NM

**Figure 2:**
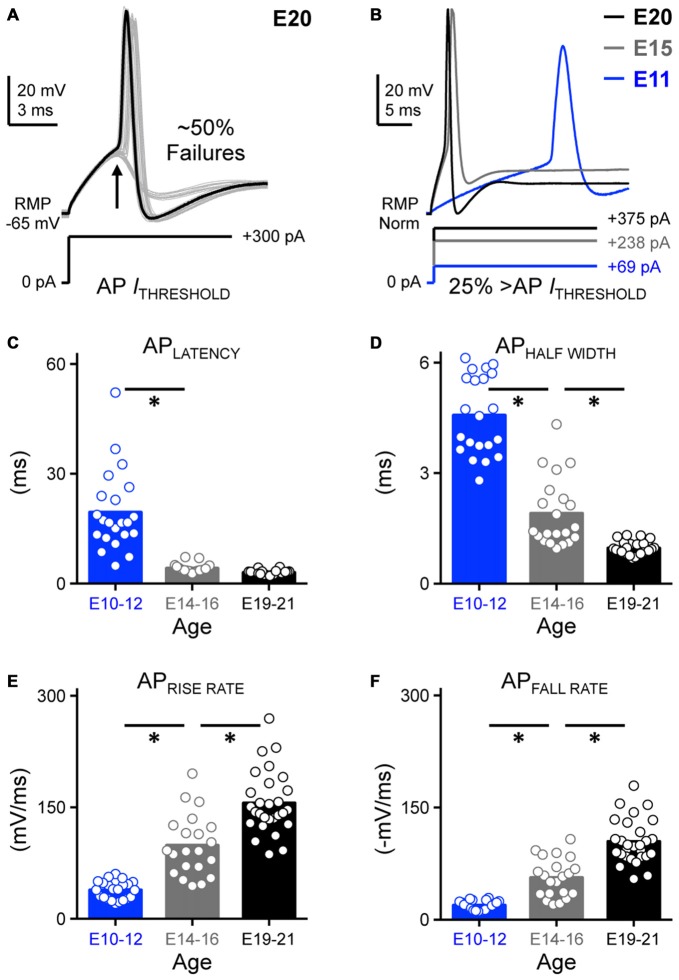
**Development of action potential kinetics in NM. (A)** Representative voltage traces (30 superimposed) in response to sustained threshold current injections. Threshold current is defined as the minimum amount of current required for NM neurons to generate an AP ~50% of the time across 30 repetitive stimulations. The injected +300 pA current shown below the traces was determined as the threshold current for this E20 NM neuron. Arrow depicts ~50% AP failures. **(B)** Representative APs for each age group evoked by sustained current injections at strength 25% above threshold current. AP kinetics were calculated and plotted as a function of age and are shown in **(C–F). (C–F)** Population data showing developmental changes in AP latency **(C)**, AP half width **(D)**, AP rise rate **(E)**, and AP fall rate (in absolute value, **F**) as a function of age. Open circles represent an individual neuron and solid bars represent the average for each age group. *p < 0.05, Bonferroni adjusted *t*-test.

We next characterized the development of basic AP properties in response to prolonged injections at suprathreshold current levels. Threshold current is defined as the minimal current required for NM neurons to respond with APs ~50% of the time across 30 stimulation trials (interpulse stimulus intervals = 2 s). The threshold current for the E20 neuron shown in Figure 2A was +300 pA and the sustained current yielded ~50% failures across 30 presentations (*arrow*). Once threshold current was determined for each individual neuron, we injected a sustained current command (duration = 100 ms) 25% above threshold current to characterize and compare AP properties across each age group.

Voltage responses (Figure 2B) generated by sustained suprathreshold current commands were used to quantify two major AP properties: AP kinetics and reliability. Four underlying variables were analyzed with respect to AP kinetics: latency, half width and maximal rise/fall rates. Latency was defined as the time of peak AP occurrence relative to the onset of injected current. Half width was quantified as AP duration measured at half of the maximum amplitude relative to the RMP. Rise and fall rate was calculated as the maximum rate of increase and decay in the AP depolarizing and repolarizing phase, respectively. We observed that the latency of AP generation was relatively stable from E14 and did not significantly vary in the time of peak occurrence between the E14–16 and E19–21 age groups (*p* > 0.99, Figures 2B,C, Table 1). However, APs generated from early developing neurons (E10–12) occurred significantly later in time compared to the later developing populations (*p* < 0.0001, Figures 2B,C, Table 1). In parallel with a significant reduction in AP half width (*p* < 0.0001. Figure 2D, Table 1), both the AP maximal rise (Figure 2E) and fall rates (in absolute value, Figure 2F) became dramatically larger with age (*p* < 0.0001, Table 1). Changes in these four variables demonstrate that AP generation in NM neurons became significantly faster with development.

**Figure 3:**
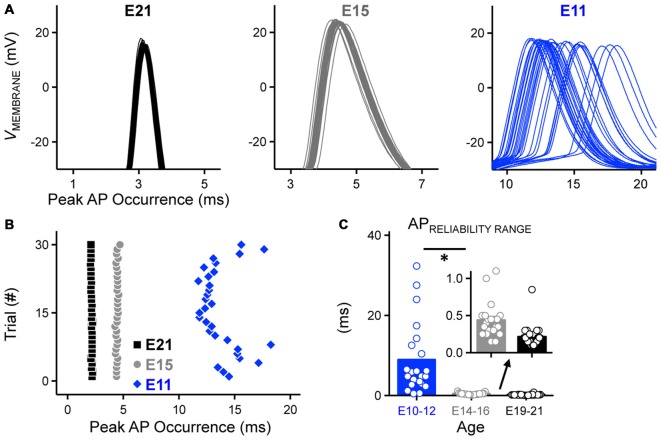
**Development of AP reliability in NM. (A)** Partial representative AP traces (30 superimposed, interpulse stimulus interval = 2 s) showing the time of peak AP occurrence from NM neurons at E21 (*left*), E15 (*middle*) and E11 (*right*). Corresponding time points of AP peak are plotted as individual dots in the same time scale for all three age groups shown in **(B). (B)** Representative raster plot showing peak AP occurrence. **(C)** Population data showing changes in the range of peak AP occurrence as a function of age. Inset showing the enlarged scale of the range for E14–16 (*left*) and E19–21 (*right*). Open circles represent an individual neuron and solid bars represent the average for each age group. *p < 0.05, Bonferroni adjusted *t*-test.

In order to quantify AP reliability, we stimulated NM neurons using suprathreshold current levels across 30 trials (interpulse stimulus intervals = 2 s). Using this protocol we were able to estimate AP reliability of individual neurons, defined as the range of time points of peak AP occurrence. Only the E11 neuron shows a visible range in peak AP occurrence and this range of peak AP occurrence decreased with age (Figures 3A–C). Across the population of neurons tested for each age group, AP reliability range was lowest for late developing neurons and although after E16, range continued to decrease slightly (i.e., reliability improved) with maturation, it was not significantly different for the two older groups (*p* > 0.99, Figures 3B,C *inset*, Table 1). There was however a nearly 40-fold increase in AP range for early developing neurons (Figures 3A–C, Table 1), demonstrating that the reliability of AP generation improved remarkably by hearing onset.

Increasing the recording temperature can greatly change the physiological condition of neurons. To examine the effect of temperature on AP properties in NM, we recorded APs generated before and after increasing the recording temperature to near physiological conditions (~35°C). At E19–21 we observed a significant increase in AP kinetics at higher temperatures. For example, AP rise rate increased from 149.00 mV/ms to 185.10 mV/ms (*p* < 0.001). In addition, AP reliability also improved significantly at higher temperatures (Supplementary Figure 1A). In a subset of experiments we also recorded APs from E19–21 NM neurons using a low-pass filter cut-off of 5 kHz, to examine whether AP kinetics of late developing neurons were underestimated when the filter cut-off was set to 2 kHz. Indeed, AP kinetics improved slightly, albeit significantly, with the higher low-pass filter cut-off frequency (Supplementary Figure 1B). In summary, late developing NM neurons generate faster and more reliable APs when recorded at near physiological temperatures and a lower low-pass filter cut-off frequency underestimate AP kinetics of late developing neurons.

**Figure 4:**
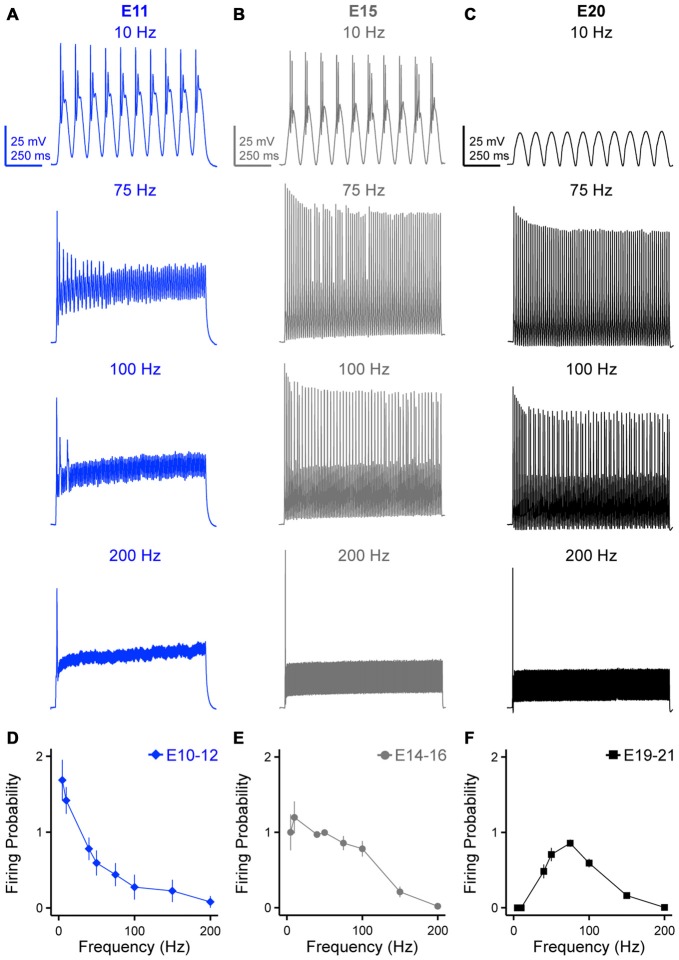
**Developing NM neurons show distinct firing patterns in response to sinusoidal current injections at varying frequencies. (A–C)** Representative voltage traces recorded from NM neurons at E11 **(A)**, E15 **(B)** and E20 **(C)** in response to 10, 75, 100 and 200 Hz sinusoidal current injections. The strength of sinusoidal current is 150% above threshold current. **(D–F)** Firing probability per sinusoidal cycle, calculated as the number of APs divided by the total number of sinusoidal cycles, is plotted as a function of stimulus frequency for NM neurons at E10–12 **(D)**, E14–16 **(E)** and E19–21 **(F)**. Error bars = standard error.

Time-coding auditory brainstem neurons fire synchronized APs by “locking” to a specific phase of incoming afferent inputs. Previous research has shown that the firing rate of time-coding auditory brainstem neurons are able to reliably follow frequency inputs as high as 400 Hz with precise fidelity (Wang et al., 1998; Gao and Lu, 2008). However, these studies used square pulse current injections that do not account for synaptic kinetic because of the abrupt onset of the pulse. In order to account for some variability in synaptic kinetics across development (e.g., presynaptic release probability, time of activation/inactivation and desensitization of postsynaptic receptors), we injected suprathreshold sinusoidal currents at frequency of 5, 10, 40, 50, 75, 100, 150 and 200 Hz to obtain a better idea of NM neurons firing probability per sinusoidal cycle. The strength of injected currents was 150% above threshold current for each neuron to ensure AP generation across trials. Representative responses for 10, 75, 100 and 200 Hz at each age group are shown in Figures 4A–C. AP generation was distinguished from passive membrane oscillations by voltage heights relative to RMP that were within two standard deviations below the average AP heights reported in Table 1. Firing probability per sinusoidal cycle (for simplicity, “firing probability”) was calculated as the number of APs divided by the total number of sinusoidal cycles and plotted as a function of stimulus frequency. All NM neurons across the three age groups only fired a single AP at the onset of the highest stimulation frequency (i.e., 200 Hz, Figures 4A–C, *bottom traces*), which shared similarities with the voltage responses to sustained current injections. For early developing neurons (E10–12), we observed a low-pass filter-like firing pattern (Figure 4A *top trace* and Figure 4D). These early developing neurons fired more than one AP within each sinusoid cycle in response to stimuli of 5 and 10 Hz. (firing probability at 5 Hz = 1.69; firing probability at 10 Hz = 1.42). As the stimulus frequency became higher, the firing probability dropped dramatically (Figure 4D), likely due to slow AP kinetics at this age. In contrast, we observed a band-pass filter-like firing pattern for late developing neurons (E19–21, Figures 4C,F). Neurons in this age group fired optimally in response to stimuli of 75 Hz (firing probability = 0.86), but less reliably to lower frequency stimulation (i.e., 40 and 50 Hz). More strikingly, they did not generate any APs at stimulus frequencies of 5 or 10 Hz. As the stimulus frequency increased above 75 Hz, the firing probability dropped gradually as well (Figure 4F). In addition and unlike E10–12 neurons, none of the late developing neurons fired more than one AP per sinusoidal cycle. NM neurons at E14–16 showed a pattern of firing probability that resembled a combination of both the early and late developing age groups (Figures 4B,E).

In order to better understand more about the frequency firing probability pattern of late developing neurons (E19–21), in a subset of experiments we systematically injected low frequency (5 and 10 Hz) sinusoidal currents at levels that exceeded nearly 1000% above AP threshold current. Surprisingly in these experiments, no NM neuron fired APs to low frequency stimulation regardless of the current injection level (Supplementary Figure 2). This suggests that late developing NM neurons behave as steady band-pass filter neurons. This result is likely due to an increase in K_V_ conductances and Na_V_ channel inactivation properties at this age (discussed further below).

### Development of Passive Membrane Properties in NM

**Figure 5:**
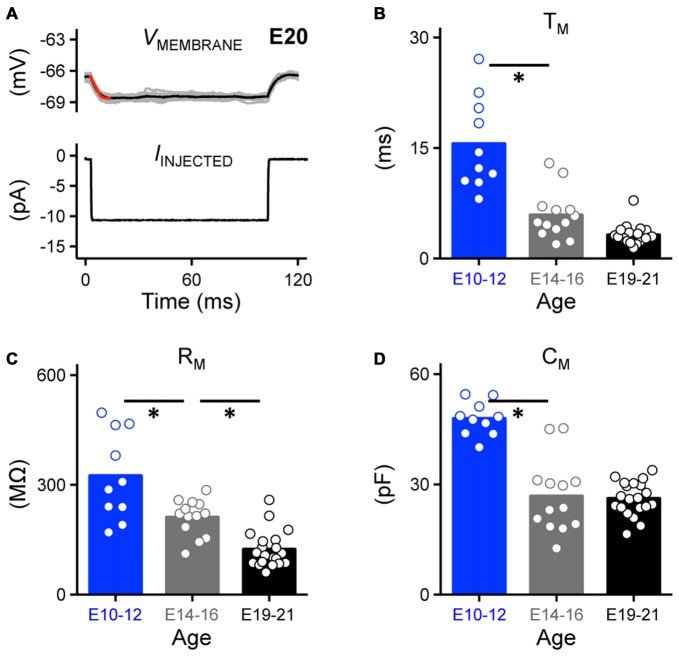
**Development of passive membrane properties in NM. (A)** Representative voltage traces recorded from an E20 NM neuron (upper, 30 superimposed) in response to a small hyperpolarizing current (lower, −10 pA). A single exponential was fit to a 10 ms time window following the current injection (superimposed red line), in order to calculate passive membrane properties shown in **(B–D). (B–D)** Population data showing developmental changes in membrane voltage time constant (T_M_, **B**), membrane input resistance (R_M_, **C**) and membrane capacitance (C_M_, **D**) as a function of age. Open circles represent an individual neuron and solid bars represent the average for each age group. *p < 0.05, Bonferroni adjusted *t*-test.

Passive membrane properties are highly relevant to AP kinetics and reliability and may partially account for the probability of frequency firing patterns of NM neurons. In order to better understand how these properties developmentally regulate AP generation in NM neurons, we characterized the development of the membrane voltage time constant, membrane input resistance and membrane capacitance. Under whole-cell current clamp, we first characterized the passive intrinsic properties for each age group by applying a small hyperpolarizing somatic current injection (−10 pA, Franzen et al., 2015, Figure 5A, *bottom*). The membrane voltage time constant (tau, T_M_) and steady-state voltage responses were quantified by fitting a single exponential to a 10 ms time window of the voltage response following the initial current injection (superimposed red line, Figure 5A, *top*). The membrane input resistance (R_M_) was obtained by dividing the calculated steady-state voltage response to the injected current. Membrane capacitance (C_M_) was calculated based on the equation C_M_ = T_M_/R_M_. Across the population of neurons tested for each age group, the time constant became significantly faster with development; there was a nearly 4-fold reduction from E10 to E21 (*p* < 0.0001, Figure 5B, Table 1). Although the time constant continued to shorten after E14, it was not significantly different between the E14–16 and E19–21 age groups (*p* = 0.12, Figure 5B). The membrane input resistance became significantly smaller with development (*p* < 0.0001, Figure 5C, Table 1), likely due to a developmental increase in K_V_ conductances. The membrane capacitance also decreased with age (Figure 5D, Table 1), however, membrane capacitance of E14–16 and E19–21 age groups was comparable (*p* > 0.99, Figure 5D). This developmental change in membrane capacitance suggests a significant reduction in neuron size likely due to the dramatic pruning of dendrites known to occur during the development of NM neurons and that this pruning is nearly established by E14 (Jhaveri and Morest, 1982a,b).

### Development of Voltage Dependent Potassium Channels in NM

**Figure 6:**
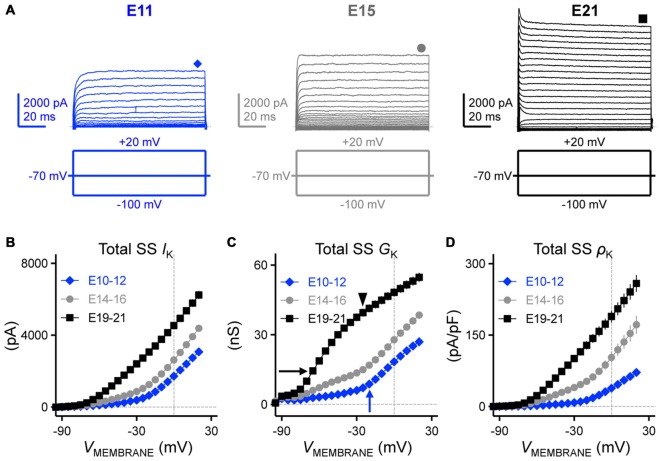
**Developing NM neurons showed a significant increase in total steady-state K_V_ currents. (A)** Representative K_V_ current traces (*I*_K_) recorded from NM neurons at E11 (*left*), E15 (*middle*) and E21 (*right*) in response to membrane voltages clamped from −100 to +20 mV (voltage step = 5 mV, voltage duration = 100 ms). Symbols (diamond, circle and square) at the end of current traces represent time window of measured steady-state K_V_ currents (SS *I*_K_). **(B–D)** Population data showing the relationship of total steady-state K_V_ currents (SS *I*_K_, **B**), conductance (SS *G*_K_, **C**) and density (SS ρK, **D**) to the varying membrane voltages for each age group. Arrows and arrowhead in **(C)** indicate the apparent activation of distinct K_V_ channel subtypes at different ages. Note that data points at −100 mV are not shown for simplicity.

In mature time-coding auditory brainstem neurons, K_V_ channels are important in regulating AP properties (Rathouz and Trussell, 1998; Wang et al., 1998; Scott et al., 2005; Klug and Trussell, 2006). In line with these studies, we hypothesized that the refinement of K_V_ channels plays an important role in shaping AP properties in developing NM. In order to test this hypothesis we first characterized the development of K_V_ channel currents by holding neurons across a range of voltages (Figure 6A). Average steady-state K_V_ currents were measured from a 10 ms time window at the end of the voltage command and plotted as a function of membrane voltage for each age group (Figure 6B). We observed a significant increase in the total amount of steady-state K_V_ currents with development; at the membrane voltage command of +20 mV there was a 2-fold increase from E10 to E21 (*p* < 0.0001, Figure 6B, Table 1).

We next addressed what factors accounted for the increase in total K_V_ currents. Such factors include a developmental upregulation in the expression of K_V_ channels—as well as—increases in the conductance of individual K_V_ channels. In order to identify which mechanism contributes to the increase in total K_V_ currents, we calculated K_V_ channel conductance and channel density and plotted them as a function of membrane voltage (Figures 6C,D, respectively). We observed a significant increase in both K_V_ channel conductance (Figure 6C) and K_V_ channel density (Figure 6D) across the three age groups (at +20 mV, *p* < 0.0001, Table 1). These results revealed that the combinatory effect of larger K_V_ channel conductance and higher K_V_ channel density resulted in a significant increase in the total K_V_ currents. Moreover, for late developing NM neurons (E19–21) there are two inflectional points in their conductance-voltage relationship that reveal different slope trajectories (black arrow and arrowhead in Figure 6C), indicating the apparent activation of two distinct K_V_ channel subtypes, most likely to be K^+^_LVA_ (Figure 6C, black arrow) and K^+^_HVA_ channels (Figure 6C, black arrowhead). This observation was not as clear for E14–16 NM neurons despite a considerable amount of K_V_ channel conductance at more negative membrane voltages than −40 mV. However, for E10–12 NM neurons only one inflectional point was observed (Figure 6C, blue arrow). This is likely due to the distinct ontogeny of different K_V_ channel subtypes (see below).

**Figure 7:**
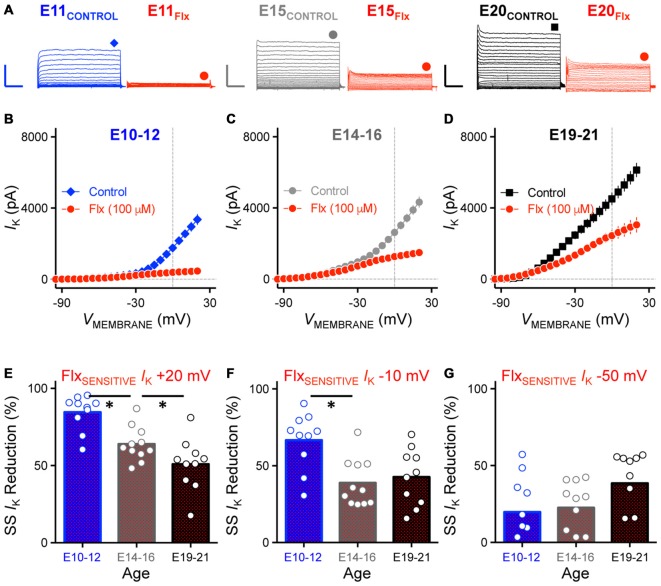
**Reduction of K^+^_HVA_ current with Flx application was developmentally regulated. (A)** Representative K_V_ current traces recorded from NM neurons at E11 (*left*), E15 (*middle*) and E20 (*right*) before and during fluoxetine (Flx, 100 μM) application, in response to membrane voltages clamped from −100 to +20 mV. Flx is a potent K_V_3.1-containing K^+^_HVA_ channel blocker. Scale bar values are 2000 pA/20 ms. Data obtained during drug application are shown in red. Symbols (diamond, circle and square) at the end of current traces represent time window of measured steady-state K_V_ currents. **(B–D)** Population data showing current-voltage relationship before and during Flx application for E10–12 **(B)**, E14–16 **(C)** and E19–21 **(D)**. Note that data points at −100 mV are not shown for simplicity. **(E–G)** Population data showing reduction in steady-state K_V_ currents at membrane voltage of +20 **(E)**, −10 **(F)** and −50 **(G)** mV measured as percent change and plotted as a function of age. Note that in **(G)** several negative data points are not shown in the figure (2 points for E10–12, 1 for E14–16 and 1 for E19–21). Open circles represent an individual neuron and solid bars represent the average for each age group. *p < 0.05, Bonferroni adjusted *t*-test.

To test whether there is a developmental difference in the maturation of K_V_ channel subtypes in NM, we first bath applied Flx (100 μM), a highly potent blocker for K_V_ channels that contain the K_V_3.1 subunit, the major subunit of K^+^_HVA_ channels that are expressed in many time-coding auditory brainstem neurons (Parameshwaran-Iyer et al., 2001, 2003; Lu et al., 2004; Bortone et al., 2006). For each age group we observed a differential reduction in the amount of K^+^_HVA_ current with Flx application that was voltage dependent (Figure 7A). Across the population of NM neurons tested, the current-voltage relationship for the Flx-insensitive current is shown in Figures 7B–D for each age group. For E10–12 NM neurons, 84.56% of the steady-state K_V_ current was reduced after Flx application when the membrane voltage was held at +20 mV. This percent reduction gradually decreased to 63.88% and 50.84% for E14–16 and E19–21 neurons, respectively (*p* < 0.001, Figure 7E, Table 2). Similarly, when the membrane voltage was held at −10 mV, the percent reduction was 66.64%, 38.81% and 42.66% for E10–12, E14–16 and E19–21 neurons, respectively (*p* < 0.01, Figure 7F, Table 2). In contrast, only 19–38% of the total steady-state K_V_ current was reduced when the membrane voltage was held at −50 mV from E10 to E21 neurons (*p* = 0.10, Figure 7G, Table 2). The reduction at −50 mV was comparable across all three age groups and is likely due to Flx being a non-potent blocker for Kv1.1-containing K^+^_LVA_ channels. Previous pharmacological study shows that Flx at this concentration only blocks ~25% Kv1.1 mediated K_V_ currents (Tytgat et al., 1997). The results at +20 and −10 mV suggest that for early developing NM neurons, the majority of K_V_ current is high-voltage activated. There was only a negligible amount of Flx-insensitive current remaining, which is likely mediated by K^+^_LVA_ channels at E10–12. Therefore, only one inflectional point was observed in conductance-voltage relationship for early developing NM neurons (see Figure 6C). Furthermore, because the ratio of K^+^_HVA_ current relative to the total K_V_ currents dropped to 49.16% for E19–21 neurons suggests that there are comparable amounts of K^+^_HVA_ and K^+^_LVA_ currents in late developing NM neurons. Thus, the two inflectional points shown in Figure 6C likely represent the separate activations of these two functionally distinct K_V_ channel subtypes.

**Table 2:** **Change in potassium channel properties with different blockers**.

	E10–E12	E14–E16	E19–E21	ANOVA *P*
**Percentage change in Kv current (*I_k_*) with bath application of Flx^∧^ (%)**
*I*_k_ reduction at −50 mV	19.74 ± 23.03 (10)	22.55 ± 16.65 (11)	38.46 ± 20.96 (10)	*P* = 0.11 (Figure 7G)
*I*_k_ reduction at −10 mV	66.64 ± 18.40 (10)	38.81 ± 15.37 (11)	42.66 ± 18.48 (10)	*P* < 0.01 (Figure 7F)
*I*_k_ reduction at +20 mV	84.56 ± 11.35 (10)	63.88 ± 11.10 (11)	50.84 ± 16.80 (10)	*P* < 0.001 (Figure 7E)
Percentage change in action potential (AP) properties with bath application of TEA^*^ (%)
AP half width increase	178.90 ± 143.30 (9)	67.27 ± 22.53 (12)	45.41 ± 39.40 (20)	*P* < 0.0001 (Figure 9B)
Max fall rate reduction	66.73 ± 10.69 (9)	46.51 ± 10.92 (12)	37.98 ± 17.08 (20)	*P* < 0.001 (Figure 9C)
Threshold current reduction	41.41 ± 24.45 (9)	33.68 ± 10.01 (12)	21.97 ± 11.49 (20)	*P* < 0.01 (Figure 9D)

Flx^∧^, Fluoxetine (100 μM), Kv3.1-containing $K_{HVA}^{+}$ channel blocker. TEA*, Tetraethylammonium (1 mM), Kv3.1–3.4 blocker.

**Figure 8:**
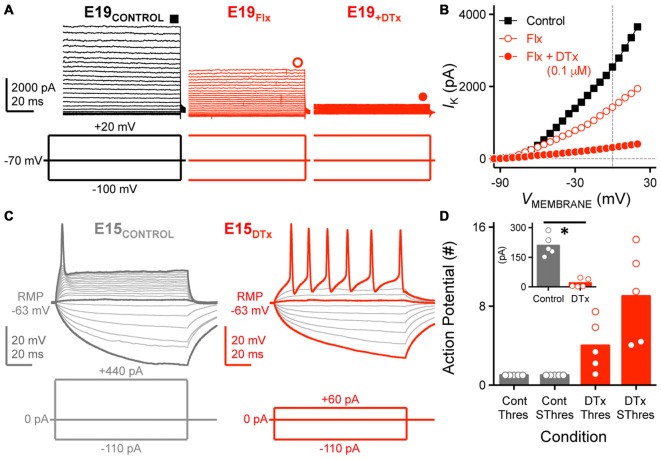
**K^+^_LVA_ channels mediate the majority of Flx-insensitive K_V_ current and regulate neural excitability. (A)** Steady-state K_V_ current traces recorded from an E19 NM neuron before (control) and during subsequent bath application of Flx and DTx (0.1 μM). DTx is a potent K_V_1.1, K_V_1.2-containing K^+^_LVA_ channel blocker. Data obtained during drug application are shown in red. Symbols (square and circles) at the end of current traces represent time window of measured steady-state K_V_ currents. **(B)** Current-voltage relationship before and during subsequent drug application for the E19 NM neuron shown in **(A)**. Note that data points at −100 mV are not shown for simplicity. **(C)** Representative voltage traces recorded from an E15 NM neuron before and during DTx application, in response to a sequence of sustained current injections (current step = 20 pA, current duration = 100 ms). **(D)** Population data showing the number of APs recorded from NM neurons at E14–16 and counted under different conditions: control (Cont) or during DTx application (DTx), in response to threshold (Thres) or suprathreshold injected current (SThres). Inset is the population data showing a significant reduction in threshold current before and during DTx application. Open circles represent an individual neuron and solid bars represent the average for each age group. *p < 0.05, Bonferroni adjusted *t*-test.

To test this idea, we sequentially bath applied DTx (0.1 μM) for a subset of E19–21 NM neurons. DTx is a potent and selective blocker of K_V_1.1 and K_V_1.2-containing K^+^_LVA_ channels. Bath application of Flx, the K^+^_HVA_ channel blocker, reduced the maximum steady-state current by ~50% for the E19 neuron shown in Figure 8A (*middle trace*, and Figure 8B) as previously shown (see Figures 7A,D). The remaining current was nearly abolished with subsequent bath application of DTx (Figure 8A *right trace*, and Figure 8B) suggesting that K^+^_LVA_ channels mediated a large majority of the Flx-insensitive current.

Numerous studies have also shown that K^+^_LVA_ channels regulate neural excitability and prevent mature NM neurons from firing multiple APs to strong and sustained depolarization (Rathouz and Trussell, 1998). The role of K^+^_LVA_ channels in early developing neurons is largely unexplored. However, because E10–12 neurons have relatively small amounts of K^+^_LVA_ current (see Figure 7) and can fire multiple APs to sustained depolarization current steps (see Figure 1), we instead tested whether K^+^_LVA_ are responsible for controlling neural excitability for E14–16 neurons. When K^+^_LVA_ channels were blocked, neural excitability was elevated for this age group (Figures 8C,D). In the control condition (Figure 8C *left trace*), a strong sustained depolarizing current injection (+440 pA) generated only a single AP at the onset of the stimulation, a firing phenotype similar to late developing NM neurons. In the presence of DTx, the neuron’s rheobase was reduced such that a relatively small depolarizing current injection (+60 pA) elicited multiple APs throughout the duration of the depolarizing stimulation (Figure 8C *right trace*). These results—reduced rheobase, reduced AP threshold current and increased AP excitability—were consistent across the population of neurons tested (Figure 8D) and suggest that K^+^_LVA_ channels have a strong contribution in regulating neural excitability as early as hearing onset in NM (Howard et al., 2007).

### Developmental Regulation of AP Properties by K^+^_HVA_ Channels in NM

**Figure 9:**
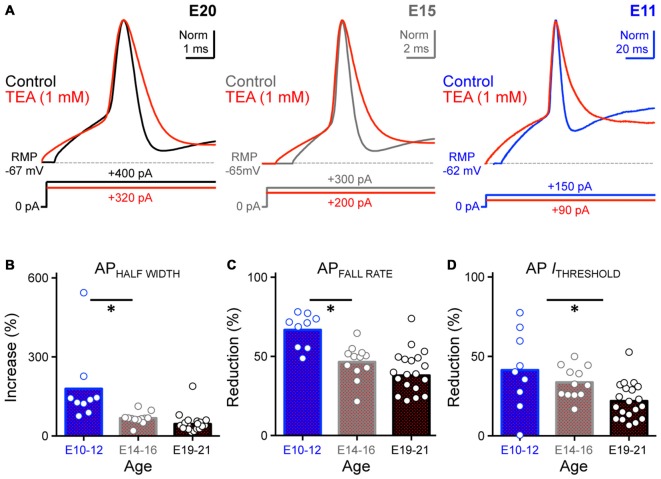
**Blockade of K^+^_HVA_ channels altered AP kinetics of developing NM neurons. (A)** Normalized representative APs recorded from NM neurons at E20 (*left*), E15 (*middle*) and E11 (*right*) before (control) and during bath application of low-concentration TEA (1 mM). Data obtained during drug application are shown in red. Sustained current injections are 25% above threshold current. **(B–D)** Population data showing percent changes in AP half width **(B)**, fall rate **(C)** and threshold current **(D)** during TEA application, as a function of age. Open circles represent an individual neuron and solid bars represent the average for each age group. *p < 0.05, Bonferroni adjusted *t*-test.

Previous studies have shown that K^+^_HVA_ channels regulate AP kinetics, increasing the rate of repolarization and thus promoting the firing of fast APs in mature time-coding auditory brainstem neurons (for review, see Rudy and McBain, 2001; Johnston et al., 2010). Blocking these channels increases the AP half width and reduces the fall rate. However, the function of K^+^_HVA_ channels in early developing NM neurons is largely unexplored. Moreover, although we found that the ratio of K^+^_HVA_ current relative to the total steady-state K_V_ currents decreased as a function of age, how this change correlates to the function of K^+^_HVA_ channels at different ages remains unknown. In order to characterize the role of K^+^_HVA_ channels in regulating AP properties during development, we applied low-concentration TEA (1 mM) to block K^+^_HVA_ channels. Voltage responses used in data analysis before and during TEA application were evoked using injected currents that were 25% above the AP threshold current for each neuron. Consistent with previous studies in late developing auditory brainstem neurons, AP half width of E19–21 NM neurons increased significantly by an average of ~45% after blockade of K^+^_HVA_ channels (Control: 0.94 ms, TEA: 1.36 ms; *p* < 0.0001; Figure 9A *left trace*, Figure 9B, Table 2). In addition, AP fall rate and threshold current significantly decreased by an average of ~38% and ~22%, respectively (AP_FALL RATE_ = control: 105.00 mV/ms, TEA: 63.82 mV/ms; *p* < 0.0001, AP *I*_THRESHOLD_ = control: 322.30 pA, TEA: 249.00 pA; *p* < 0.0001, Figures 9C,D, Table 2). The above results were consistent for the E14–16 age group as well. AP half width significantly increased by an average of ~67% (Control: 1.69 ms, TEA: 2.84 ms; *p* < 0.001, Figure 9A *middle*, Figure 9B, Table 2), and AP fall rate and threshold current significantly decreased by an average of ~47% and ~34%, respectively (AP_FALL RATE_ = control: 64.78 mV/ms, TEA: 33.04 mV/ms; *p* < 0.0001, AP *I*_THRESHOLD_ = control: 215.90 pA, TEA: 143.60 pA; *p* < 0.0001, Figures 9C,D, Table 2).

Because the ratio of K^+^_HVA_ current is greatest at E10–12 (see Figure 7), we speculated that the largest changes in AP properties during TEA application would occur for early developing NM neurons. Indeed, we found that for E10–12 neurons the AP half width significantly increased by an average of ~180% when K^+^_HVA_ channels were blocked (Control: 3.65 ms, TEA: 10.39 ms; *p* < 0.01, Figure 9A *right*, Figure 9B, Table 2). Similarly, AP fall rate and threshold current significantly decreased by an average of ~67% and ~42% respectively (AP_FALL RATE_ = control: 24.61 mV/ms, TEA: 10.80 mV/ms; *p* < 0.0001; AP *I*_THRESHOLD_ = control: 156.00 pA, TEA: 91.13 pA; *p* < 0.001, Figures 9C,D, Table 2). Taken together, these results suggest that K^+^_HVA_ channels differentially regulate AP kinetics in developing NM neurons and that this difference is reflected in the relative current amounts of K^+^_HVA_ for each age group. Furthermore, differential contributions of K_V_ channels are also developmentally regulated and play an important role in shaping AP properties in developing NM neurons with the most pronounce refinement occurring during and after the onset of hearing (E14–21, respectively).

### Development of Voltage Dependent Sodium Channels in NM

For decades, much effort has been devoted to the role of K_V_ channels in the auditory system. However, Na_V_ channels are also an important component for AP generating of time-coding auditory brainstem neurons, but surprisingly, the early developmental properties of Na_V_ channels have been largely unexplored. To determine the extent to which Na_V_ channel currents differ with maturation, we characterized their developmental profile in NM neurons.

**Figure 10:**
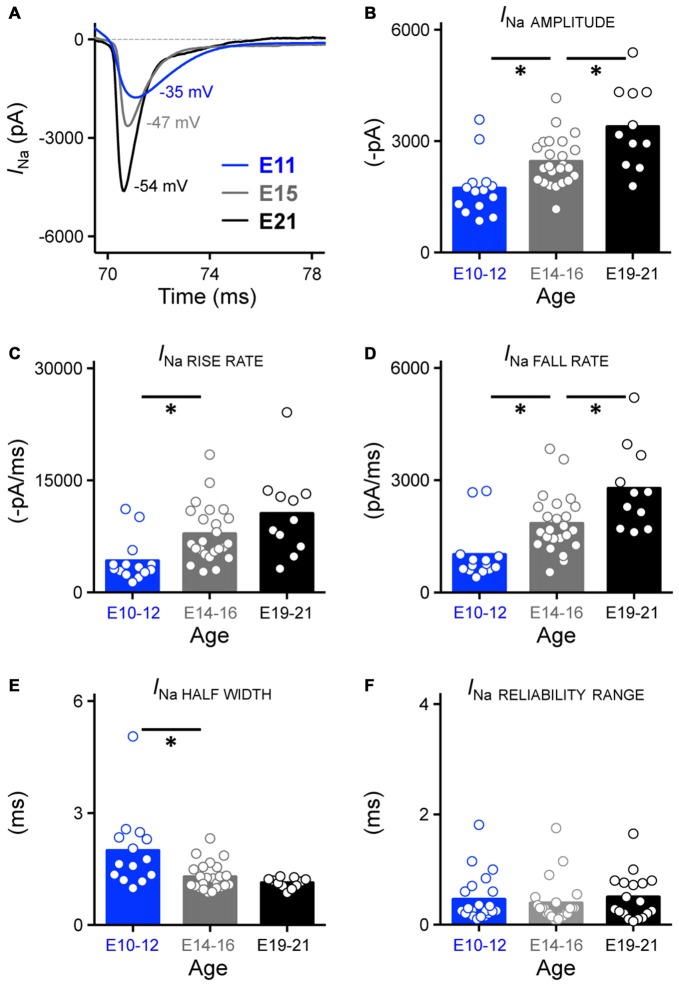
**Development of Na_V_ current properties in NM. (A)** Representative Na_V_ current traces (*I*_Na_) recorded from NM neurons at E11, E15 and E21 in response to membrane voltages clamped at 25% above channel activation voltage (−35, 47 and −54 mV for E11, E15 and E21 NM neuron in this figure, respectively). These currents were used to measure the sodium current properties for each age group shown in **(B–F). (B–F)** Population data showing developmental changes in Na_V_ current amplitude (in absolute value, **B**), rise rate (in absolute value, **C**), fall rate **(D)**, half width **(E)** and reliability range **(F)** as a function of age. Open circles represent an individual neuron and solid bars represent the average for each age group. *p < 0.05, Bonferroni adjusted *t*-test.

Isolated Na_V_ currents were recorded at a membrane voltage 25% above their activation voltage and K_V_ channels were blocked with bath application of 4-AP and TEA in a normal concentration of ACSF (see “Materials and Methods” Section). Using this protocol we characterized Na_V_ channel current amplitude, kinetics and reliability and compared these variables at each age group (Figure 10A).

Across the population of neurons tested, we found that the amplitude of isolated Na_V_ currents significantly increased with development, with nearly a 2-fold growth between E10–21 (*p* < 0.0001, Figure 10B, Table 1). We also observed significant changes in Na_V_ channel kinetics. The rise and fall rate of Na_V_ currents, which indicate how fast the ion channel activates and inactivates, became significantly larger with development (*p* < 0.01, Figures 10C,D, Table 1). In parallel with these changes, the half width of Na_V_ currents became significantly smaller (*p* < 0.01, Figure 10E, Table 1) but reliability of Na_V_ channels remained constant across development (Figure 10F, Table 1), suggesting that the precision of Na_V_ channels is established before hearing onset. We also recorded from a subpopulation of NM neurons at E10–12 (*n* = 8) and E19–21 (*n* = 9) with digitization set at 50 kHz instead of 20 kHz. Since no difference of Na_V_ reliability was found between the two sampling rates and developmental similarities remained constant, these data are reported together in Figure 10F. In a subset of experiments we examined whether Na_V_ channel kinetics of late developing neurons were underestimated due to our low-pass filter cut-off of 2 kHz. Indeed, Na_V_ channel kinetics improved slightly, albeit significantly, with a higher low-pass filter cut-off of 5 kHz (Supplementary Figure 3A). Therefore, it should be noted that lower low-pass filter cut-off frequency underestimated Na_V_ channel kinetics of late developing neurons.

**Figure 11:**
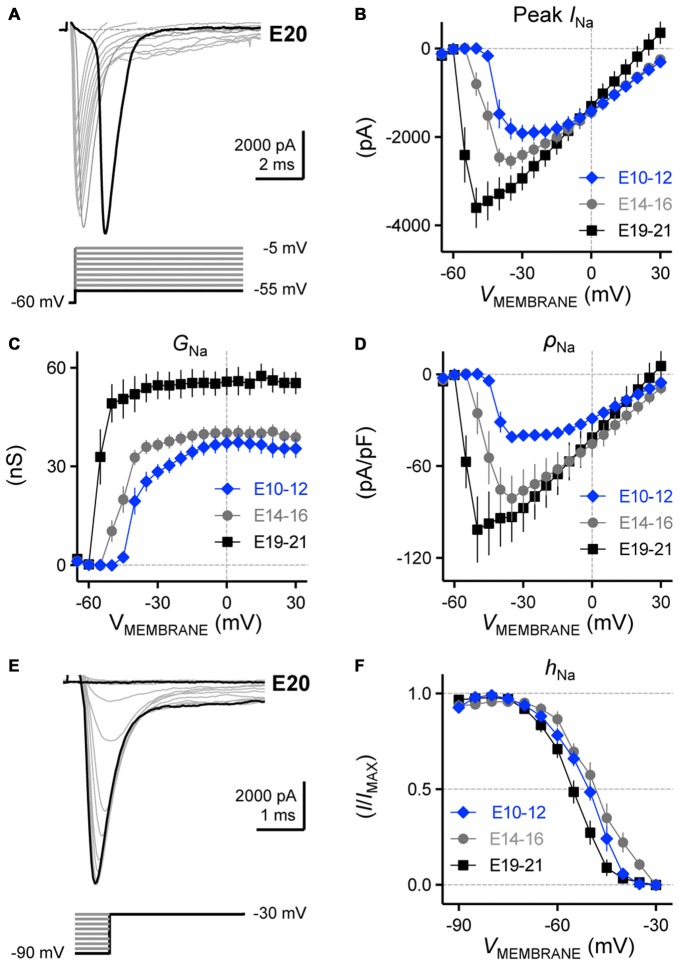
**Voltage dependent Na_V_ current activation and inactivation in developing NM neurons. (A)** Representative Na_V_ current traces recorded from an E20 NM neuron in response to membrane voltages clamped from −55 to −5 mV (voltage step = 5 mV, voltage duration = 100 ms). **(B–D)** Population data showing the relationship of peak Na_V_ current (peak *I*_Na_, **B**), conductance (*G*_Na_, **C**) and density (*ρ*_Na_, **D**) to the varying membrane voltages for each age group. **(E)** Representative Na_V_ current traces recorded from an E20 NM neuron in response to depolarization to −30 mV following pre-pulse holding voltages clamped from −90 to −30 mV (voltage step = 10 mV). **(F)** Population data showing voltage dependence of Na_V_ channel inactivation for each age group. *h*_Na_ was calculated as the Na_V_ current recorded for each trial normalized to the maximum current across all trials and plotted as a function of the pre-pulse holding voltage. Error bars = standard error.

Next, we determined if there were developmental changes in the voltage dependence of Na_V_ channel activation in NM neurons and representative traces from an E20 neuron are shown in Figure 11A. When plotted across the entire voltage range tested, we observed a significant increase in maximum Na_V_ current and a developmental shift in Na_V_ voltage dependence (*p* < 0.0001, Figure 11B, Table 1). From E10 to E16, the voltage dependent change in Na_V_ current shown in Figure 11B is consistent with an increase in current density but not channel conductance. In contrast, from E16 to E21, Na_V_ channel conductance became considerably larger with minimal variation in current density compared to early developing neurons (Figures 11C,D, respectively).

When recording isolated Na_V_ currents, a major concern is voltage-clamp errors that result from large Na_V_ currents and their extremely fast activation phase (Cummins et al., 2009). To improve the quality of voltage clamp, some previous studies used low-Na^+^ ACSF, reducing the driving force for sodium ions and thus resulting in smaller and slower Na_V_ current currents (Lin, 1997; Kuba and Ohmori, 2009; Kuba et al., 2010). To examine the possibility of voltage-clamp errors in our study, we recorded from a subpopulation of NM neurons across the three age groups in low-Na^+^ ACSF (see “Materials and Methods” Section) and characterized Na_V_ current amplitude and kinetics (Supplementary Figure 3B). Na_V_ current amplitude was reduced by ~50% for all three age groups, in line with ~50% reduction in the external sodium concentration. More importantly, Na_V_ current amplitude showed a similar developmental trend in larger amplitudes (E10–12 = −874.10 pA, E14–16 = −1464.00 pA, E19–21 = −1569.00 pA). We also observed similar developmental changes in Na_V_ channel kinetics, for example, rise rate was 2525 pA/ms for NM neurons at E10–12, which increased to 4055 pA/ms and 4777 pA/ms at E14–16 and E19–21, respectively. In addition, the general shape of current-voltage relationships for all three age groups remained similar (Supplementary Figure 3C). Taken together, we were able to determine that in our study the voltage-clamp errors possibly caused by normal concentration ACSF were minimal. With concern that low-Na^+^ ACSF alters gating properties of Na_V_ channels (Cummins et al., 2009), we used normal ACSF as the bath solution in subsequent experiments.

The inactivation of Na_V_ channel current is frequency dependent in other sensory neurons (Rush et al., 2005), which likely affects AP repolarization and firing precision but its developmental profile in NM is unknown. We examined the developmental changes in voltage dependence of Na_V_ inactivation. In order to study this property, NM neurons were depolarized to −30 mV using a sustained voltage pulse. This depolarizing voltage pulse followed pre-pulse holding voltages that ranged from −90 to −30 mV and representative traces from an E20 neuron are shown in Figure 11E. We determined the ratio of Na_V_ inactivation (*h*_Na_) by calculating the Na_V_ current recorded for each trial normalized to the maximum current across all trials and plotted as a function of the pre-pulse holding voltage. We found that E10–12 and E14–16 neurons have Na_V_ channels with very similar voltage dependence profiles (*V*_1/2_ *p* = 0.74, Figure 11F, Table 1). In contrast—and despite variations in Na_V_ channel amplitude, kinetics, conductance and density—E19–21 neurons contain Na_V_ channels that inactivate at more negative membrane voltages than early developing neurons. At a slightly depolarizing holding voltage of −55 mV, only ~30% of Na_V_ channels at E10-E16 were inactivated while this ratio increased to ~51% for E19–21 neurons (*p* < 0.05). Taken together, the functional development of Na_V_ current amplitude, kinetics, reliability, voltage dependence and inactivation properties in NM are in agreement with developmental changes in Na_V_ channel subtypes in NL, as previously reported by immunohistochemical experiments (Kuba et al., 2014).

## Discussion

In this study, we report the developmental profile of ion channel specializations in the avian NM at three distinct developmental time periods, corresponding to before, during and after hearing onset. We found that several factors shape developmental differences in the generation of fast and reliable APs. Our results show significant developmental changes in AP properties and frequency firing probability patterns in response to square and sinusoidal stimulation, respectively. The development of passive membrane properties increases AP kinetics and the neuron’s ability to follow higher frequencies. These changes in AP and passive membrane properties are the result of specific developmental alterations in K_V_ and Na_V_ ion channels.

### K_V_ Channel Subtypes Function Differentially in Shaping AP Properties During NM Development

Distinct auditory regions in all vertebrates accurately encode temporal properties of acoustic information (Carr and Soares, 2002; Grothe et al., 2010). Avians and mammals share analogous auditory regions that process temporal information at the cellular, synaptic and neural network level (Carr et al., 2001; Köppl, 2009). An excellent example of fast and reliable encoding occurs in the mammalian AVCN and the analogous avian NM. Unlike traditional high-frequency hearing mammalian research models, such as mice and rats, chickens utilize cues provided by both low- and high-frequency signals to encode auditory information (Hyson, 2005). With respect to hearing, chickens are also precocious animals; that is, their auditory system is near functional maturation at birth (E21) and the onset and refinement of hearing occurs during embryonic stages (Rubel et al., 1976; Rubel and Fritzsch, 2002; Jones et al., 2006). Our results regarding the development of AP properties in NM neurons support this idea from an electrophysiological perspective. Early in embryonic development and prior to hearing onset (<E12), NM neurons generate APs with slow kinetics, poor reliability and limited capacity to follow sinusoidal inputs. During embryonic development, APs become faster and more reliable such that by E14–16, APs properties are comparable to late developing NM neurons (>E19).

The significant improvement in AP kinetics is due to the development of K_V_ conductances, specifically from current mediated by K^+^_HVA_ channels. Although multiple studies have shown that blocking K^+^_HVA_ channels in mature neurons decreases AP speed (for review, see Rudy and McBain, 2001), the function of these channels in early developing auditory brainstem neurons (<E12) remains largely unexplored despite evidence of their presence as early as E10 (Parameshwaran-Iyer et al., 2003). The current study helps fill this gap by demonstrating that before hearing onset in NM, K^+^_HVA_ channels dramatically regulate AP kinetics. These channels continue to function throughout the developmental process but the strength of this regulation decreases as a function of age. In the current study, the largest changes in AP kinetics were observed at E10–12 when K^+^_HVA_ channels were blocked. Our voltage clamp results show that the majority of K_V_ currents at E10–12 are K^+^_HVA_ dependent (~85% at +20 mV, ~67% at −10 mV). In contrast, the proportion of K^+^_HVA_ current decreases greatly from E14 to E21, likely due to the rapid and dramatic development of other Kv channel subtypes, more specifically from currents mediated by K^+^_LVA_ channels. These observations suggest that different Kv channel subtypes start developing at different ages with K^+^_HVA_ channels appearing as early as E10–12 and other Kv channel subtypes developing at later ages.

Previous electrophysiology studies in NM and other auditory brainstem structures demonstrated that the development of K^+^_LVA_ channels begins around hearing onset (Howard et al., 2007; Gao and Lu, 2008), and the amount of K^+^_LVA_ current increases rapidly after the initiation of K^+^_LVA_ channel development (Scott et al., 2005). Our findings in NM neurons are consistent with these studies. In our youngest embryos, K^+^_LVA_ current only accounts for ~15% of the total K_V_ currents at +20 mV, and ~33% at −10 mV. A significant increase in the amount of K^+^_LVA_ current occurs after E14. By the time of hatch, the amount of K^+^_LVA_ current is comparable to that of K^+^_HVA_ current (~48% at +20 mV and ~57% at −10 mV). The development of K^+^_LVA_ channels is also highly influential on the developmental changes in firing properties of NM neurons. Studies in mature neurons showed that K^+^_LVA_ channels control neural excitability; large outward flux of K^+^ ions near rest prevents NM neurons from firing multiple APs in response to a sustained depolarizing current injection (Rathouz and Trussell, 1998). Similarly, at E14–16, we observed a large increase in neural excitability when K^+^_LVA_ channels were blocked. Therefore, the multiple APs we observed at E10–12 are likely due to the underdevelopment of K^+^_LVA_ channels at this age.

### Interplay of K^+^_HVA_ and K^+^_LVA_ Channels Shape the Development of Frequency Firing Probabilities in NM

In this study we applied a stimulation protocol that required injecting sinusoidal currents of varying frequencies into NM neurons. The rise time of sinusoidal current injection is not as steep as that of current pulse trains, which have been used in previous studies (Lin, 1997; Wang et al., 1998; Gao and Lu, 2008). Therefore, we explored different frequency firing probabilities of neurons at various ages in response to a range of stimulation frequencies. Using this protocol we found that E10–12 NM neurons act as a low-pass filter, responding with multiple APs to each cycle of the sinusoid at very low frequencies (5 and 10 Hz), but with poor probability to sinusoidal frequencies higher than 40 Hz. In contrast, late developing NM neurons (E19–21) act as a band-pass filter; firing optimally within the frequency range of 40–100 Hz, and surprisingly, do not generate APs in response to 5 or 10 Hz stimulation (regardless of the magnitude of the injected current). These observations are likely to be the reflection of the combinatory development of K^+^_HVA_ and K^+^_LVA_ channels.

Although the majority of K_V_ currents at E10–12 are K^+^_HVA_, the total amount is still much lower than late developing neurons. Therefore, AP repolarization is slow, which sets the upper frequency limit for NM neurons (<E12) to follow higher frequencies. In contrast, for late developing neurons (>E19), a large amount of K^+^_HVA_ current allows neurons to follow stimulus with relatively high probability up to 100 Hz. Indeed, when we blocked K^+^_HVA_ channels at this age, a dramatic reduction in firing probability was observed for 100 Hz and 150 Hz (~20% and ~60% reduction, respectively, Supplementary Figure 4). However, we observed only a slight decrease in firing probability for 75 Hz (~8% reduction, Supplementary Figure 4), suggesting that other factors might be involved in shaping the firing probability patterns for NM neurons at this frequency. We speculate that one factor involves the activation of K^+^_LVA_ channel. As discussed in more detail below, the development of K^+^_LVA_ channels shortens the time constant of NM responses to current stimulation. Thus, during blockade of K^+^_HVA_ channels, late developing NM neurons can still fire APs at 75 Hz with relatively high probability.

We hypothesize that the large disparity of response to very low frequency sinusoidal stimulation between early and late developing NM neurons is also due to developmental changes in K^+^_LVA_ channels. The rise of the low-frequency sinusoidal stimulus is long and slow; upon membrane depolarization, a large number of K^+^_LVA_ channels in late developing neurons open and repolarize the membrane before the activation of Na_V_ channels can occur. Therefore, low-frequency stimuli fail to drive neurons to fire APs at E19–21. In contrast, due to the lack of K^+^_LVA_ channels at E10–12, NM neurons are highly excitable to slow rise-time stimulation. To test this hypothesis, we used DTx to block K^+^_LVA_ channels for late developing neurons. We found that neural excitability increased dramatically after blockade of K^+^_LVA_ channels. Spontaneous and evoked activity was superimposed such that it was difficult to assess factors that regulate this response property. Future modeling experiments would help resolve this issue by changing the relative ratio of K_V_ and Na_V_ channel densities and conductances during different low frequency stimulation protocols.

### Contribution of Passive Membrane Properties to the Development of AP Generation in NM

Our results for passive membrane properties revealed a significant reduction in both the time constant and input resistance, which is consistent with previous findings in other developing auditory brainstem structures (Scott et al., 2005; Gao and Lu, 2008; Kuba et al., 2014; Franzen et al., 2015). Developmental changes in time constant and input resistance help regulate AP generation. Either during the AP depolarizing or repolarizing phase, the current flow through the membrane changes the membrane potential. A shorter time constant indicates a faster response of the membrane potential to the current, and thus improves the speed of APs. As neurons mature, the time constant typically becomes shorter, resulting in a developmental increase in AP speed.

A decrease in input resistance requires a greater magnitude of current injection to reach AP threshold for mature auditory brainstem neurons. We found that the threshold current for AP generation is significantly larger for late developing NM neurons. The reduction in input resistance is due to an increasing number of open ion channels while at rest. Our results demonstrate that a prominent increase in K^+^_LVA_ current from E14 to E21 likely contributes to the change in input resistance. From the current-voltage relationship of late developing NM neurons we report that a greater proportion of K^+^_LVA_ channels are activated near the RMP. The opening of these channels at E19–21 significantly reduces input resistance. Thus, the developmental increase in K^+^_LVA_ current shortens the time constant of NM neurons, which leads to a faster AP generation and better probability in following higher frequency sinusoidal stimulation.

We also found a significant decrease in membrane capacitance. Membrane capacitance is proportional to the surface area of the neuron, and can be used as an index of neuron size. Previous anatomical studies have shown that NM neurons begin embryonic development with dendritic-like processes that extend away from the soma (Jhaveri and Morest, 1982a,b). Conversely, later in embryonic development, most if not all dendrites are pruned away while the soma of the neuron increases in size (Rubel et al., 1976; Rubel and Fritzsch, 2002). The observed decrease in membrane capacitance suggests that the loss of surface area, caused by a dramatic pruning of dendrites, outweighs the enlargement of the soma. Thus, the total surface area of the neuron decreases and from a functional perspective, this process changes dramatically from E10–14.

### Development of Na_V_ Channel Properties in NM

In addition to K_V_ channels, significant developmental changes in Na_V_ channel kinetics contribute to increased AP speed. Na_V_ channels activate and inactivate much faster with age. Surprisingly, the generation of peak Na_V_ current is remarkably reliable for early developing neurons with little or no change in precision as a function of maturation. These findings suggest that Na_V_ channels in NM neurons develop extremely fast kinetics and the fact that AP speed improves significantly with age is reflected in the development of Na_V_ channel properties. However, AP reliability is poor in early developing NM neurons. We speculate that immature passive membrane properties and K^+^_LVA_ channels are likely the source of poor AP reliability early in NM development. As mentioned above, NM neurons <E12 have a very slow time constant, due to a lack of K^+^_LVA_ current. Although Na_V_ channels are highly reliable at early developing neurons, they still require a larger time window to charge the membrane and generate an AP. This likely increases the chance of poor reliability, AP interruption and/or noise during a long charging process. With development, NM neurons express an upregulation in K^+^_LVA_ channels in order to shorten the membrane time constant and assist Na_V_ channels in improving AP reliability.

The maximum amplitude of Na_V_ currents increased in two distinct developmental patterns. From E10 to 16 the current density of Na_V_ channels, which indicates the number of Na_V_ channels per unit area, increased significantly with minimal change in individual channel conductance. In contrast, from E16 to E21 there was a significant increase in Na_V_ channel conductance that was accompanied by developmental differences in voltage dependence and activation/inactivation properties. The changes in ion channel conductance and voltage dependence suggest a developmental switch in Na_V_ channel subtypes, especially from Nav1.2 to Nav1.6, which has been shown in the visual system (Boiko et al., 2003). Supportive evidence of this hypothesis also comes from separate characterization of Nav1.2 and Nav1.6 channels in mouse spinal sensory neurons (Rush et al., 2005). Na_V_1.2 channels are present with more depolarizing activation and inactivation voltages, and greater accumulation of inactivation at high frequency stimulation. This biophysical property helps generate lower frequency AP firing compared to the Na_V_1.6 channels, which promote higher frequency AP firing. Interestingly, a previous immunohistochemistry study in NL (the downstream output of NM) reported that early developing NL neurons contain Na_V_1.2 channels that are replaced by Na_V_1.6 channels in late developing neurons, which is considered a major subtype component for mature Na_V_ channels elsewhere in the nervous system (Caldwell et al., 2000; Black et al., 2002; Eijkelkamp et al., 2012). In NL, the developmental transition from Na_V_1.2 to Na_V_1.6 occurs around E18, which is within the time window of the functional changes in the Na_V_ conductance and voltage dependence reported here. These findings suggest that NM and NL may have similar developmental profiles of Na_V_ channel subtypes but further immunohistochemistry or pharmacological studies in NM are warranted.

### Developmental Changes in Active and Passive Properties Parallel the Development of Presynaptic Activity and Postsynaptic Properties

Previous research on the development of avian cochlear ganglion neurons (i.e., auditory nerve) shows that rudimentary responses to airborne sound first appear at E14, with tremendous refinement occurring after E16 (Jones et al., 2001, 2006). As such, E14–16 is referred to as the period of “hearing onset” and any time point before as the “prehearing period” (Jones et al., 2006). As early as E14 in this study, NM neurons are able to generate a single AP at the beginning of prolonged current depolarization. This developmental time period corresponds to hearing onset and their voltage responses resemble the mature-like phenotype. Although some developmental differences still exist between E14–21, this AP response profile is unlike early developing NM neurons before hearing onset (E10–12). Moreover, E14–16 NM neurons are capable of generating APs whose kinetics and reliability are comparable to late developing neurons. This was partially due to a dramatic shortening in their membrane voltage time constant, which reduced ~3 fold compared to early developing neurons. The rapid development of K^+^_LVA_ channels starts around hearing onset and accounts for the decrease in the membrane voltage time constant. Parallel to this development, Na_V_ channels promote stronger and faster depolarizing and inactivating currents while K^+^_HVA_ channels regulate the speed of AP repolarization for E14–16 NM neurons. Taken together, these results indicate that after E12, AP properties of NM neurons undergo substantial developmental refinement that starts as early as E14, where they begin to demonstrate a more mature-like functional phenotype. This corresponds to the onset of hearing in avians and resembles the functional development of the auditory periphery (Jones et al., 2006).

Moreover, the different firing probability to sinusoidal inputs we observed with maturation suggests that the activity of developing NM neurons resembles the changing patterns of input they receive from the developing auditory periphery. The input from cochlear ganglion neurons is mostly a combination of spontaneous and evoked activity after E14 (Jones et al., 2006). However, before hearing onset, the auditory periphery spontaneously generates the activity that NM neurons receive at E10–12 in the absence of sound (i.e., endogenous signaling). This spontaneous activity is shaped by bursts of APs followed by long periods of silence—a very low-frequency rhythm that differs fundamentally from the quasi-Poisson activity of the mature periphery (Lippe, 1994; Jones and Jones, 2000; Jones et al., 2001). Interestingly, early developing NM neurons in our study respond optimally with bursts of AP firing to low-frequency stimuli, but poorly to frequencies higher than 40 Hz.

At E14–16, cochlear ganglion neurons provide spontaneous and evoked activity across a broad range of frequencies (Jones et al., 2006). Our results in NM are in agreement with these findings at E14-E16; neurons can fire relatively reliable APs in response to sinusoidal stimulation between 5–100 Hz. This frequency response range is wider compared to late developing NM neurons (40–100 Hz). These data suggest that E14–16 NM neurons have similar low frequency firing probability patterns compared to early developing neurons but also begin to show mature-like responses compared to higher frequency stimulation, accommodating both spontaneous and evoked peripheral input across a broader range of frequencies.

With maturation, bursts of spontaneous activity are replaced by consistent steady-state activity, which appears in the auditory periphery around E19. In addition, cochlear ganglion neurons are able to respond to acoustic stimulation reliably with narrow and mature-like tuning curves (Rebillard and Rubel, 1981; Jones et al., 2006). Comparable with these aforementioned developmental changes in spontaneous and evoked activity, E19–21 NM neurons in our study did not respond to very low-frequency stimuli and preferentially respond to stimuli within a narrow frequency range (40–100 Hz). It should be noted that late developing NM neurons responded optimally to evoked sinusoidal stimulus at 75 Hz. This is higher than the spontaneous firing rate of cochlear ganglion neurons previously reported for the same developmental time period (e.g., 20 Hz) but almost identical to that reported from hatchlings (e.g., 74 Hz, Jones and Jones, 2000). Taken together, our current findings suggest a developmental relationship of spontaneous and evoked activity between peripheral and central auditory neurons. Developing NM neurons differentially regulate their AP firing pattern to sinusoidal stimulation. This process appears to be dependent on the developmental interplay between K_V_ and Na_V_ channels, suggesting a parallel maturation of the spontaneous and evoked activities NM neurons receive from their afferent inputs.

This parallel development of evoked AP firing properties of NM neurons and cochlear ganglion neurons, as well as previous studies focusing on peripheral and central activity in the auditory system, suggests that peripheral activity may have a neurotrophic effect on the refinement of intrinsic ion channel properties of central relay neurons (Levi-Montalcini, 1949; Parks, 1979; Rubel and Fritzsch, 2002; Wang and Bergles, 2015). However, in order to relay this activity with significant biological relevance from neuron to neuron, a reliable synaptic connection needs to be established. Before hearing onset (<E12), input from cochlear ganglion neurons elicit relatively small EPSCs in NM neurons (Lu and Trussell, 2007). To accommodate this reduced synaptic input conductance, underdeveloped K^+^_LVA_ channels enables NM neurons to fire to weak levels of presynaptic input. We speculate that the immature intrinsic properties before hearing onset are essential for establishing a stable synaptic connection between the periphery and NM neurons. After hearing onset (i.e., E19–21), presynaptic inputs are mature-like and synchronized glutamate release elicits large EPSCs in NM (Lu and Trussell, 2007). Correspondingly, increased K^+^_LVA_ expression levels—which largely accounts for the greater amount of inward current needed to generate APs—controls NM neurons excitability in order to accurately encode and filter its presynaptic input.

In summary, the developmental interplay between passive and active ion channel membrane properties, along with known changes in presynaptic activity and postsynaptic characteristics, likely ensures the development of remarkably fast and reliable AP generation. This occurs just a few days following hearing onset in the avian NM and are specializations required for sound localization and auditory temporal coding.

## Author Contributions

HH, JTS designed the study. HH performed experiments and analyzed the data. BF, LR analyzed the data and helped perform the experiments. HH, BF, LR and JTS wrote the original version of the manuscript. HH, JTS finalized the manuscript.

## Conflict of Interest Statement

The authors declare that the research was conducted in the absence of any commercial or financial relationships that could be construed as a potential conflict of interest.
