# Supplementary figures and images for: Developmental Profile of Ion Channel Specializations in the Avian Nucleus Magnocellularis
Source: Front Cell Neurosci. 2016 Mar 30;10:80. doi: 10.3389/fncel.2016.00080 (PMC4811932; doi:10.3389/fncel.2016.00080)

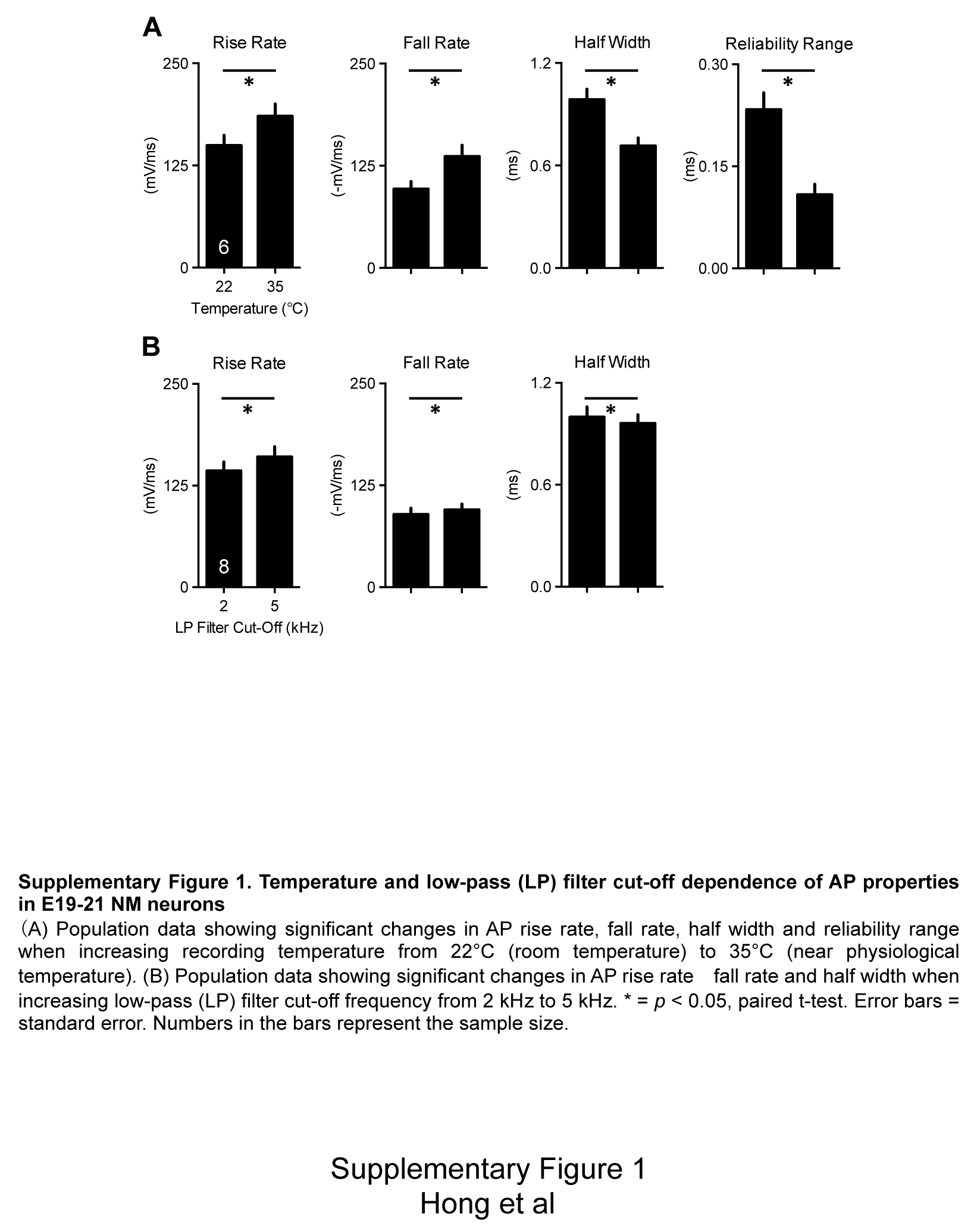

Supplement: Supplementary file 1 [file Image_1.tif]

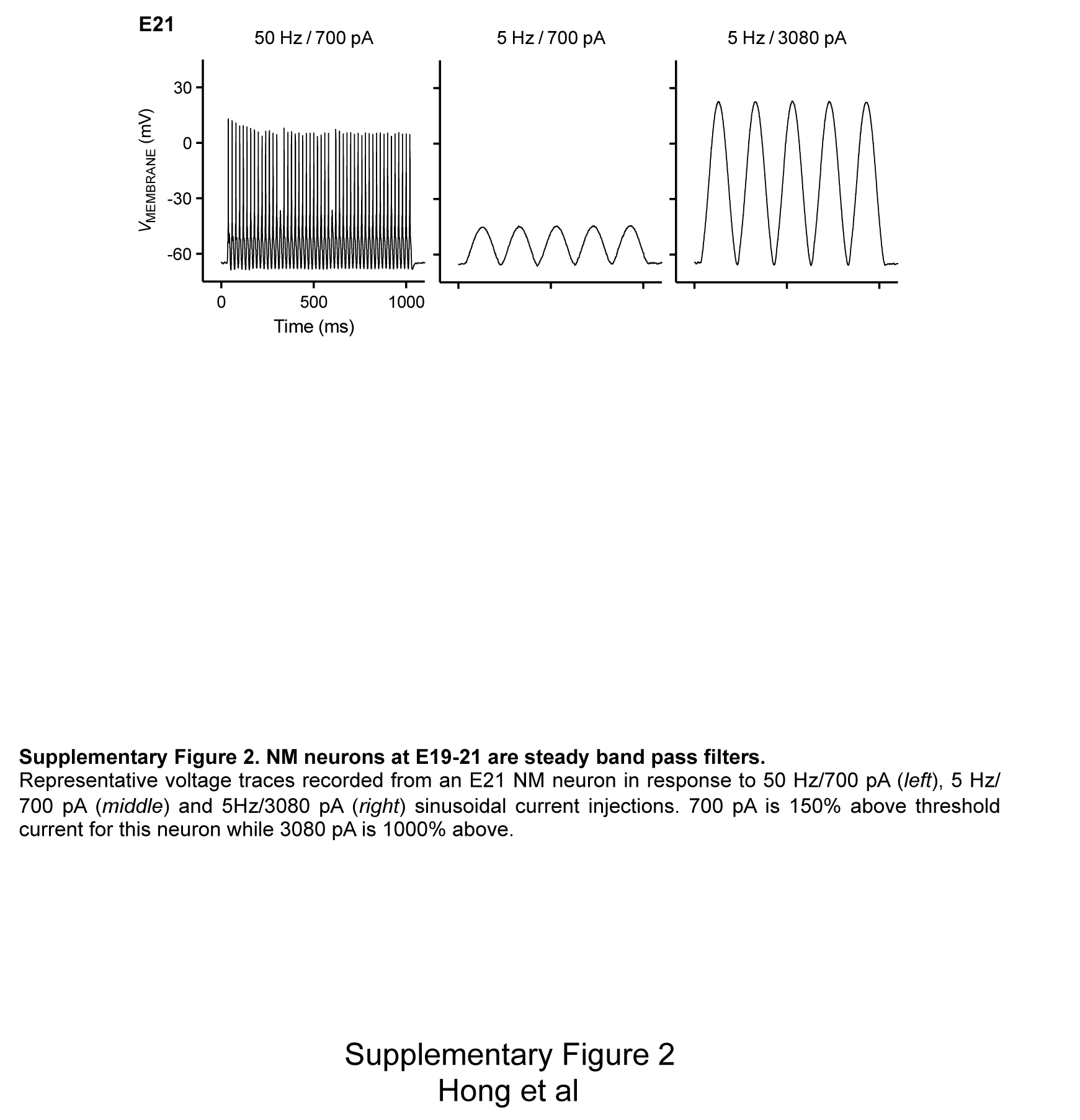

Supplement: Supplementary file 2 [file Image_2.tif]

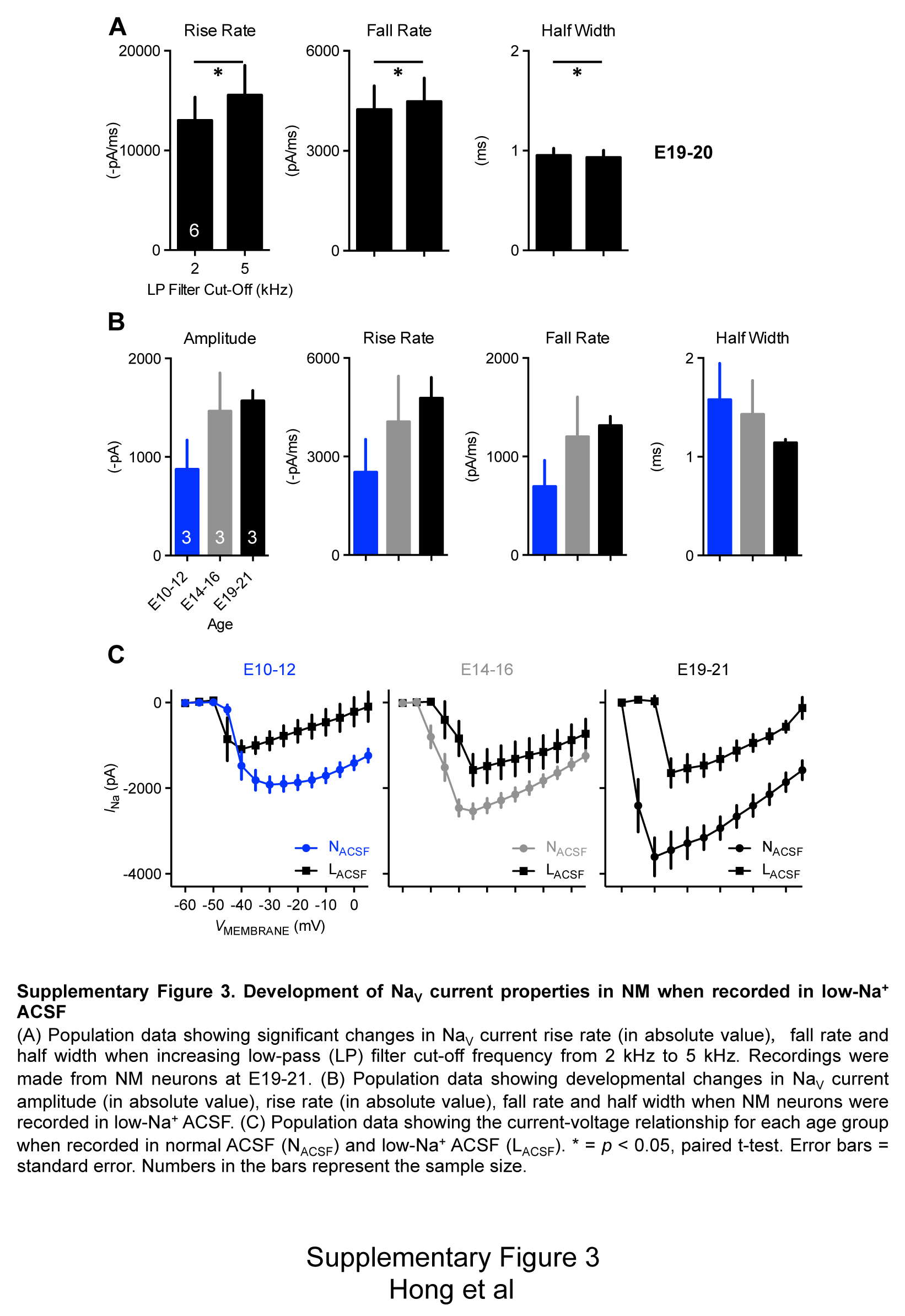

Supplement: Supplementary file 3 [file Image_3.tif]

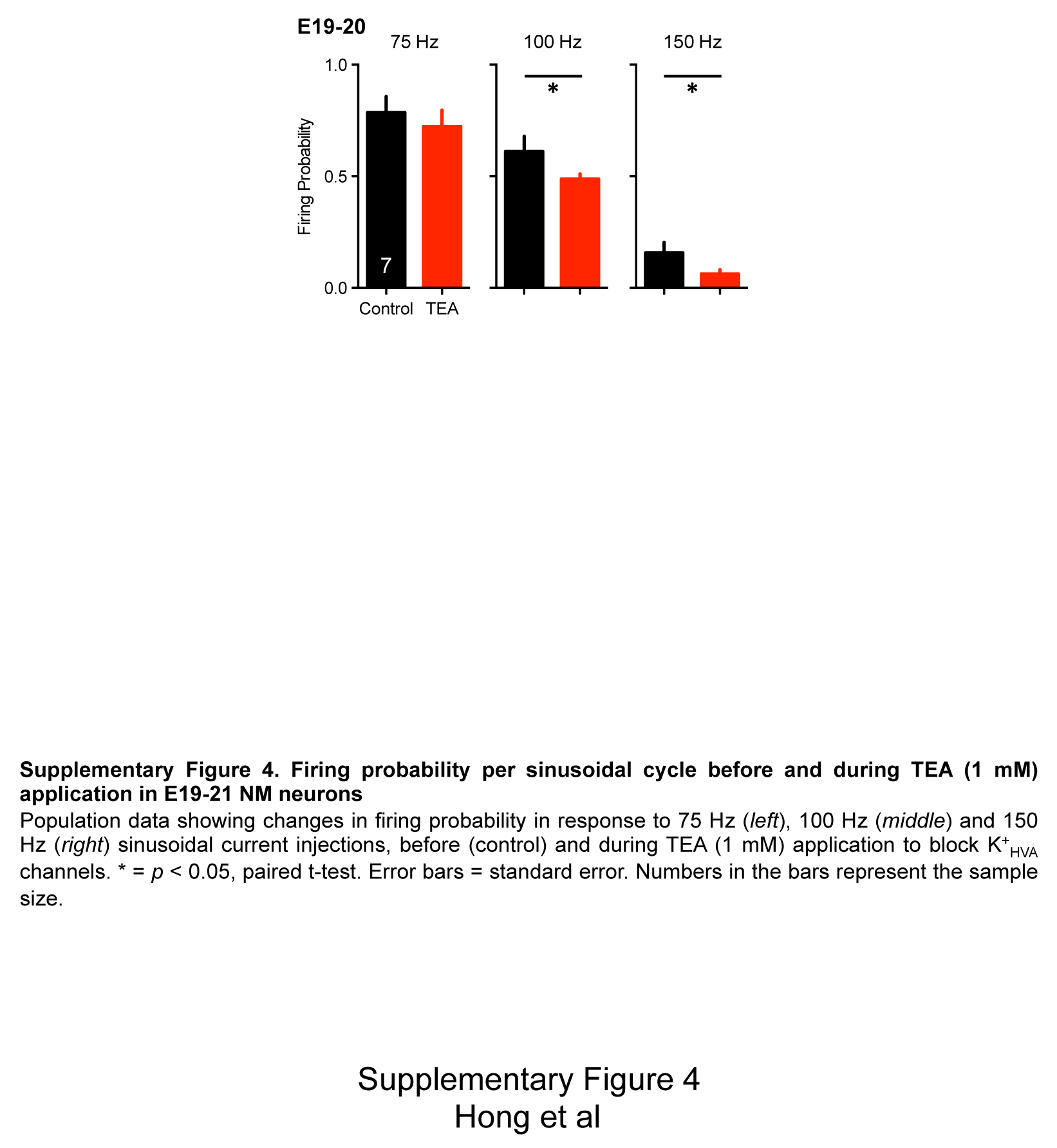

Supplement: Supplementary file 4 [file Image_4.tif]
